# Role of Microglia in Modulating Adult Neurogenesis in Health and Neurodegeneration

**DOI:** 10.3390/ijms21186875

**Published:** 2020-09-19

**Authors:** Mohammed Al-Onaizi, Alaa Al-Khalifah, Dalal Qasem, Ayman ElAli

**Affiliations:** 1 Department of Anatomy, Faculty of Medicine, Kuwait University, PO Box 24923, Kuwait City 13110, Kuwait; 2 Undergraduate Dental Program, Faculty of Dentistry, Kuwait University, Kuwait City 13110, Kuwait; alaa.alkhalifah@hsc.edu.kw; 3 Undergraduate Medical Program, Faculty of Medicine, Kuwait University, Kuwait City 13110, Kuwait; dalal.q@hsc.edu.kw; 4 Neuroscience Axis, Research Center of CHU de Québec-Université Laval, Québec City, QC G1V 4G2, Canada; 5 Department of Psychiatry and Neuroscience, Faculty of Medicine, Université Laval, Québec City, QC G1V 4G2, Canada

**Keywords:** microglia, neurogenesis, neurodegeneration

## Abstract

Microglia are the resident immune cells of the brain, constituting the powerhouse of brain innate immunity. They originate from hematopoietic precursors that infiltrate the developing brain during different stages of embryogenesis, acquiring a phenotype characterized by the presence of dense ramifications. Microglial cells play key roles in maintaining brain homeostasis and regulating brain immune responses. They continuously scan and sense the brain environment to detect any occurring changes. Upon detection of a signal related to physiological or pathological processes, the cells are activated and transform to an amoeboid-like phenotype, mounting adequate responses that range from phagocytosis to secretion of inflammatory and trophic factors. The overwhelming evidence suggests that microglia are crucially implicated in influencing neuronal proliferation and differentiation, as well as synaptic connections, and thereby cognitive and behavioral functions. Here, we review the role of microglia in adult neurogenesis under physiological conditions, and how this role is affected in neurodegenerative diseases.

## 1. Microglia

Microglia are mononuclear phagocytes that constitute the main resident immune cell population in the brain [1]. The origin of microglia has been a matter of debate; however, it is now well established that microglial cells are derived from myeloid progenitors that infiltrate the developing brain at early stages [2]. Some findings suggest that microglia originate from progenitors derived from the neuroectoderm and/or the mesoderm to invade the brain [1], while other new findings suggest that an additional pool of microglia may derive from circulating progenitors, namely the monocytes [3]. Importantly, the vast majority of microglial cell population is generated postnatally after blood–brain barrier (BBB) formation. How these cells are maintained in the adult brain during lifespan is a matter of debate. Once microglial cell progenitors have infiltrated the brain, they adopt a highly ramified morphology characterized by the presence of long and highly motile cellular processes [4]. The recent findings indicate that microglia are continuously patrolling the brain, and their motile processes act as sentinels to survey and scan the microenvironment in order to detect any occurring change in the brain homeostasis [5]. Upon the detection of a relevant biological signal, microglia rapidly get activated and modify their morphology by adopting an amoeboid-like phenotype. Depending upon the nature of the detected signals, an adapted response by activated cells is mounted, which could range from phagocytosis of biological elements to the release of various immune and non-immune molecular mediators and factors [5,6]. Importantly, under conditions in which limited neuronal damage is present, microglial cells in site actively extend their processes towards the damaged neurons, and distant cells rapidly migrate towards the damaged neurons to establish cell–cell contacts [7]. These reports clearly outline the dynamic and sophisticated multifunctional role of microglia in the brain.

## 2. Mechanisms Regulating Microglial Cell Activity

Microglia are highly dynamic and via their processes could scan the entire brain every couple of hours [8]. This requires a narrow regulation of cell processes’ movement, cell mobility, cell morphology, phagocytosis, as well as the release of non-immune and immune molecular mediators. Here, we will briefly present major mechanisms that play key roles in controlling microglial cell activity:

*(i) Pattern recognition receptors:* Microglia express several receptors that are centrally implicated in regulating the innate immunity, namely the Pattern Recognition Receptors (PRRs). PRRs comprise a subfamily of transmembrane proteins that include toll-like receptors (TLRs), nucleotide-binding oligomerization domain (nod)-like receptors (NLRs), Leucin Rich Repeats (LRRs)-containing receptors, and retinoic acid-inducible gene-1 (RIG1)-like receptors (RLRs). The initial primary identified function of these receptors is to recognize microbial components such as pathogen-associated molecular patterns (PAMPs), and danger-associated molecular patterns (DAMPs). PAMPs include bacterial and viral genetic materials, peptidoglycans, and lipopolysaccharides (LPS). DAMPs include exogenous peptidoglycans, endogenous heat shock proteins, high-mobility group box-1 (HMGB1), uric acid, adenosine triphosphate (ATP), and DNA [9]. The activation of these receptors induces a cascade of intracellular signaling pathways implicated in modulating microglial activity, namely the master regulators of inflammation, nuclear factor-kappa B (NF-κB) and interferon regulatory factor-3 (IRF3) [1].

*(ii) Cytokine receptors:* Microglia express as well a wide range of cytokine receptors and produce various cytokines including, tumor necrosis factor-α (TNFα), transforming growth factor-β (TGFβ), and interleukins (ILs). Activation of TNFα receptors (TNFRs) increases TNFα release by microglia, thus creating a positive autocrine regulatory loop that participates in microglial cell activation [10,11]. TGFβ is a multifunctional cytokine that binds to TGFβ receptor type I (TGFRI/RII) complex [12], counteracting TNFα-induced pro-inflammatory responses in microglia [13]. TGFRI/RII activation triggers the formation of Small and Mothers Against Decaplentaplegic-2/3/4 (SMAD-2/3/4) complex that regulates the expression of several inflammatory genes [12]. TGFβ reduces as well the production of IL6, interferon-γ (IFNγ), and monocyte chemoattractant protein-1 (MCP1; chemokine ligand 2 (CCL2)) [14]. Additionally, microglial cells express several IL receptors (ILRs), namely IL1Rs, IL5R, IL6R, IL8R, IL9R, IL10R, IL12R, IL13R, and IL15R. IL1β and IL6 regulate microglial cell activity by stimulating microglial pro-inflammatory responses. On the other hand, IL4 and IL10 are potent anti-inflammatory molecules that promote microglial anti-inflammatory responses [15].

*(iii) Chemokine receptors:* Chemokines are a large family of molecules that are characterized by the presence of conserved cysteine residues in their N-terminal sequences. They are classified into four distinct subgroups based on the spacing of cysteine residues, as follows, C chemokines (one N-terminal cysteine), CC chemokines (two adjacent N-terminal cysteines), CXC chemokines (one amino acid between two N-terminal cysteines), and CX3C chemokines (three amino acids between two N-terminal cysteines [16]). In the brain, chemokines are essentially released by microglia as well as neurons under specific conditions. Chemokines are produced as soluble molecules that generate a chemotactic gradient for cell migration, except for CX3C ligand 1 (CX3CL1; fractalkine), which in parallel mediates its effect as a membrane-anchored molecule [16]. Functionally, chemokines are divided into two subgroups, homeostatic chemokines that are constitutively produced contributing to basal cell migration, and inflammatory chemokines that are induced once an inflammatory response is engaged [17]. Besides being the major producer of cytokines in the brain, microglia express different types of chemokine receptors, which are G-protein coupled receptors (GPCRs), namely CCL1 receptor (CCR1), CCR2, CCR3, CCR4, CCR5, CCR6, CCR7, CXCR1, CXCR2, CXCR3, CXCR4 and CXCR5 (reviewed in [4]). Among these ligands/receptors is the CX3CL1/CX3CR1 pathway, which plays a particularly important role in governing neuron-microglia communication. CX3CL1 is produced exclusively in neurons, and binds to CX3CR1, which is exclusively expressed in microglia [18,19].

*(iv) Neurotransmitter receptors:* Several neurotransmitter receptors have been shown to be expressed in microglia, including purinoceptors, glutamate receptors, cholinergic receptors, adrenergic receptors and cannabinoid receptors [1,20]. These receptors seem to play important role in modulating the interaction between microglia and neurons. As natural receptors of nucleotides, purinoceptors play a central role in regulating microglial cell activity [21]. Upon neuronal dysfunction, the release of extracellular nucleotides by neurons, such as ATP, ADP, uridine triphosphate (UTP), and UDP, transmits a signal of alert, which is caped by microglial that mounts an adequate response [22,23]. These extracellular nucleotides are recognized by several purinoceptors, namely the metabotropic P1 adenosine receptors (GPCRs), ionotropic P2X purinoceptors (ligand-gated cationic channels), and metabotropic P2Y purinoceptors (G protein-coupled receptors) (reviewed in [4]). Activation of these receptors modulates microglial cell mobility and morphology [24,25] primarily via activation of calcium (Ca^2+^) signaling and MAP kinases pathways, leading to the release of various pro-inflammatory cytokines (reviewed in [26]). Furthermore, glutamate receptors, such as ionotropic α-Amino-3-hydroxy-5-methyl-4-isoxazolepropionic acid (AMPA) receptor, and metabotropic glutamate receptors (mGluRs) have been shown to be implicated in regulating microglial cell activity (reviewed in [20]) via regulation of NF-kB pathway as well as TNFα turnover [27,28]. The metabotropic G protein-coupled receptor gamma-aminobutyric acid (GABA_B_) type is expressed in a subpopulation of microglia [29]. Interestingly, GABA_B_ activation in LPS-induced microglial cells in vitro decreases the release of IL-6 and IL-12p240, but not TNFα or nitric oxide [29]. Moreover, GABA_A_ receptors have been demonstrated to promote microglial superoxide production [30], significantly attenuating the release of LPS-induced IL-6 and TNFα in culture [31]. These findings suggest that GABA_B_ and GABA_A_ receptors potentially stimulate a neuroprotective phenotype in microglia by differentially and selectively modulating the release of different cytokines. A large body of evidence has demonstrated the involvement of α7 nicotinic (α7nAChRs) expressed by microglia in regulating anti-inflammatory responses [32]. Activation of α7nAChRs inhibits the release of TNFα from microglia, mediated through deactivation of ERK1/2 and p38 MAP kinase signalling [33]. In comparison to other central nervous system (CNS) cell types, microglia show a significantly higher expression level of the β2 adrenergic receptor [34,35]. Evidence shows that norepinephrine (NE) and β2 adrenergic receptors play a critical role in stimulating microglial anti-inflammatory responses [34] via suppression of inducible nitric oxide synthase (iNOS), and IL-1β [36,37]. Moreover, activation of β1 adrenergic receptors, which are also expressed in microglia [38], attenuates the synthesis of the pro-inflammatory cytokines IL-6 and TNFα [39]. Finally, microglia have been shown to express the cannabinoid receptor CB2, which upon activation stimulates cell migration and inhibits the release of pro-inflammatory cytokines IL-6 and TNFα (reviewed in [40]).

*(v) TREM2:* Myeloid cells-2 (TREM2) receptor is a cell surface receptor that belongs to the immunoglobulin (Ig) superfamily, and is essentially expressed in microglia [41]. TREM2 is a transmembrane receptor that acts by interacting with the intracellular adaptor DNAX-activation protein-12 (DAP12) [41]. Association of TREM2 to DAP12 triggers tyrosine phosphorylation of the latter DAP12 within its immunoreceptor tyrosine-based activation motif (ITAM) signaling pathway. This association and subsequent phosphorylation leads to the reorganization of the cytoskeleton, production of chemokines, and stimulation of phagocytosis [42]. Interestingly, TREM2 binds to gram-positive and gram-negative bacterial components as well as to a number of ligands including anionic, zwitterionic, and myelin-associated lipids, deriving from the cell membrane of neurons and glial cells [43]. TREM2 binds as well to heat shock protein-60 (Hsp60), which enhances the phagocytic capacity of phagocytic cells including microglia [44].

*(vi) Phosphatidylserine receptors:* Phosphatidylserine (PS) is a phospholipid sequestered to the inner leaflet of plasma membrane under normal conditions, but gets exposed in apoptotic cells [45]. Recognition of PS by specialized receptors (PSRs) constitutes a major step in efficiently removing cell debris by microglia [46]. Microglial PSRs that directly bind to PS include the brain-specific angiogenesis inhibitor-1 (BAI1), T-cell immunoglobulin mucin receptor 1 (TIM1) and TIM4 [46]. PSRs can bind indirectly to PS using an intermediate adaptors, such as c-mer proto-oncogene tyrosine kinase (MerTK), and vitronectin receptors [46].The recent findings are indicating that while PSR activation enhances microglial cell phagocytosis while significantly mitigating in the release of various pro-inflammatory mediators including TNFα, IL1β, and nitric oxide (NO) [47].

*(vii) Scavenger receptors:* Scavenger receptors (SRs) are cell membrane receptors implicated in regulation of cell adhesion, and in uptake of negatively charged macromolecules as well as modified low-density lipoprotein (LDL) [48]. Microglial cells express several SRs that play an important role in modulating microglial cell function. These receptors include macrophage SR class AI (SR-AI), macrophage receptor with collagenous structure (MARCO), SR-B3 (CD36), macrosialin (CD68), and lectin-like oxidized low-density lipoprotein receptor-1 (LOX1). A wide range of molecules, including modified lipids and proteins, polyribonucleotides, polysaccharides, and anionic phospholipids, bind to SR-AI [49]. In turn, SR-AI activation triggers the production of several pro-inflammatory mediators, such as TNFα, IL1β, IL-6, and NO [50]. Activation of MARCO on the other hand triggers reorganization of microglial cell cytoskeleton, an important step during the process of phagocytosis and cell mobility [51]. CD36 belongs to the SR-B family [52] binding ligands, such as native/modified LDL, collagen, thrombospondin, oxidized phospholipids, and long-chain fatty acids [53]. Stimulation of the CD36 signaling pathway enhances microglial cell phagocytic capacity as well as production of pro-inflammatory cytokines, and chemokines [54]. CD68, which belongs to the SR-B family, is characterized by the presence of a mucin-like motif in the extracellular domain [55], and is actively involved in regulating microglial cell phagocytosis [56]. Finally, LOX1 belongs to the SR-E family, which binds and process oxidized LDL [57], and activation of LOX1 triggers activation of NF-κB signaling pathway leading to the release of various pro-inflammatory cytokines [58].

*(viii) Fc Receptors:* Fc receptors (FcRs) belong to the Ig superfamily possessing various immune functions, including activation of inflammatory cells, degranulation, and phagocytosis. All known FcR subgroups have been reported to be expressed in microglia, which upon activation modulates phagocytosis and cytokine release [59]. FcRs bind the constant domain (Fc) of Igs and are subdivided into different subclasses based on their binding to specific isotype classes and subclasses of Igs [60].

*(ix) Other Receptors:* Many other receptors implicated in immune response regulation are as well expressed in microglia cells. Among these, the sialic acid-binding immunoglobulin-type lectin-3 (Siglec-3; CD33) is a type 1 transmembrane receptor [59]. CD33 is a member of the CD33-related Siglecs, which recognize sialic acid residues of glycoproteins and glycolipids [59]. Activation of CD33 attenuates the phagocytic capacity of microglial cell [61]. Moreover, Siglec-E, a CD33-related Siglecs, was shown to recognize neural glycocalyx, and to inhibit the phagocytosis of neural debris by microglia [62]. Microglial express as well complement receptors (CRs), which are major regulators of the innate immune system [63,64]. Sigma-1 receptors (S1R), which are membrane-associated proteins recognizing endogenous monoamine molecules, potently regulate microglial immune functions [65]. For instance, administration of a potent allosteric S1R modulator, SKF83959 significantly reduces the release of NOS as well as the expression of pro-inflammatory cytokines, such as TNFα, IL1β, and iNOS in LPS-stimulated microglial cells in vitro [66]. Interestingly, these effects were reversed by administration of a selective S1R antagonist (BD1047) [66], supporting the key role of S1Rs in mediating the anti-inflammatory effects of microglia [65,67,68]. The progesterone receptor membrane component-1 (PGRMC1) interacts with S2R to regulate microglial cell activity, and modulates axonal sprouting [69,70]. Microglial express as well the CD200 (or OX2) cell membrane glycoprotein receptor (CD200R), a member of the Ig superfamily, and contributes to modulation of microglial cell function [71]. Finally, microglial cells abundantly express the**** receptor for advanced glycation end products (RAGE), which upon activation by binding to advanced glycosylation end products (AGE), S100/calgranulin family of proteins, and HMGB1, induces NF-κB signaling pathway and the subsequent release of different pro-inflammatory mediators [72].

## 3. Microglial Cell Functions

As mentioned above, adequate microglial cell activity/activation is required to maintain brain homeostasis by recognizing various types of biological signals [7,8]. Through their highly motile processes, microglia make specific and repeated cell–cell contacts with various cell types, and more intimately with neurons [8]. Microglial cell dynamic interaction with neighboring cells translates the complex functions of microglia as regulators of neuronal function under physiological as well pathophysiological conditions [73]. The overwhelming findings indicate that microglia support neuronal function via two major mechanisms, phagocytosis and biochemical interactions [1]. The process of phagocytosis is usually accompanied by the release of several anti-inflammatory mediators, growth and neurotrophic factors, associated to a reduction in the release of several pro-inflammatory cytokines [74]. Phagocytosis by microglia typically require pathogen-binding receptors such as TLRs, and cellular apoptotic-recognizing receptors such as TREM-2 [75]. Additional receptors involved in microglial phagocytosis Fc receptors, complement receptors, scavenger receptors, and others (reviewed in [42]). Although initially, microglial phagocytic capacity was supposed to be exclusively related to pathological conditions, emerging findings demonstrated that it is critically involved in physiological processes such as eliminating apoptotic cell debris within the neurogenic niches in the adult brain, as well as shaping circuit function via pruning and stabilization of dendritic spines [73,76,77]. Furthermore, microglial cells were shown to biochemically maintain neuronal function and synaptic plasticity by secreting different enzymes involved in the remodeling of the extracellular space, namely tissue-plasminogen activator (t-PA) and matrix metalloproteinases (MMPs) [78,79,80]. Taken together, these reports clearly outline the essential role of microglia in influencing neuronal structural and functional integrity in the adult brain.

The regulation of microglial cell phagocytosis is orchestrated by specialized and tightly controlled mechanisms that comprise find-me, eat-me, and digest-me steps [46,77,81]. These signals are mediated by the release of various molecules, such as nucleotides, cytokines, and chemokines to trigger microglial recruitment and activity modulation. In the find-me step, purinoceptors and chemokine receptors guide the activated microglial cells towards neurons that are emitting stress signals [18,23]. In the eat-me step, PSRs mediate the engulfment of apoptotic cells, leading to the formation of a phagocytic cup [46]. Finally, the digest-me step is characterized by the formation of a phagosome, which contains the apoptotic materials, leading to the formation of a phagolysosome (fusion of phagosomes with lysosomes) [82]. Importantly, microglia are capable as well of internalizing dysfunctional but viable neurons, a new form of cell death called “phagoptosis” that is essential for a timely removal of aged, senescent and damaged cells before generating cell debris, which could trigger an excessive inflammatory response [83]. This process is accompanied by a profound modulation of several microglial functions, namely a fine-tuned intracellular activation of reactive oxygen species (ROS)-mediated degradation of internalized targets in phagosomes, and modulation of the overall inflammatory responses (reviewed in [84]) [85].

## 4. Adult Neurogenesis

Neurogenesis is the process by which new neurons are generated. This process begins early in life during embryogenesis, and continues into adulthood and throughout the lifespan [86,87]. Neurogenesis occurs mainly in two main regions of the adult mammalian brain; the subgranular zone (SGZ) of the dentate gyrus (DG) in the hippocampus, and the subventricular zone (SVZ) of the lateral ventricle (LV) [86,87,88]. These regions are referred to as neurogenic niches due to the unique complex microenvironment critical for neural stem cell (NSC) development [89]. Adult neurogenesis requires long-lived NSCs, but their exact origin of NSCs remains unclear. However, it has been proposed that adult NSCs share the same origin with the embryonic dentate granule neurons, the neuroepithelium [90]. Additional evidence shows that these cells may originate from a population of sonic hedgehog (shh)-responsive cells in the ventral hippocampus, followed by a relocation of the descendants cells into the dorsal hippocampus to become the source of adult NSCs in the SGZ [91,92]. These cells undergo asymmetrical cell division and have the capacity for self-renewal and for generating other specialized cells through differentiation [93,94]. NSCs pass through several consequential steps before they become functionally active neurons [90]. NSCs population is represented by type 1 radial glia-like cells (RGLs); these cells then generate proliferating intermediate progenitor cells (IPCs, or type 2 cells) (Reviewed in [95]). Type 2 cells then commit to the neuronal lineage by giving rise to neuroblasts which become immature neurons and then finally mature to functional dentate granule neurons that are integrated into the hippocampal circuitry [90]. Type 1 cells are slowly dividing astrocytes-like cells; these cells express glial fibrillary acidic protein (GFAP), nestin, and the transcription factor SRY (sex determining region Y)-box 2 (Sox2) [96]. Conversely, type 2 cells are rapidly proliferating cells that express nestin and Sox2 but not GFAP (Reviewed in [97]). In the adult hippocampus, new neurons are generated in the SGZ then differentiate into mature neurons in the granular cell layer of the DG, where they are functionally integrated into the hippocampal circuitry [98]. Importantly, only a small fraction of newly born neurons survive and approximately 50% functionally integrate into the hippocampal circuitry in rodents [99], and about 40% of the newly born cells integrate and survive for less than 18 months in the olfactory bulb in rodents [100]. In humans, evidence demonstrates that there is no significant postnatal neuronal turnover in the human adult olfactory bulb, suggesting that olfactory bulb neurogenesis is probably absent in adult humans [101]. In contrary, all dentate granule neurons turnover in the adult human hippocampus [102], and around 10% of dentate granule neurons are subject to turnover in adulthood in mice [103,104]. The surviving cells in the SGZ project their dendrites to the molecular layer of the DG and send projections to CA3 pyramidal cells, becoming fully mature neurons (NeuN-positive) that integrate into the hippocampal circuitry. Newly born neurons in the SVZ migrate anteriorly to the olfactory bulb through the rostral migratory system, where they become functional local interneurons [86,105]. Adult SVZ neurogenesis has been associated with subependymal astrocytes, which function as NSCs-like cells to generate mammalian achaete scute homolog-1 (Mash1)-positive progenitors [106,107]. These cells then differentiate into doublecortin (Dcx)-positive neuroblasts before migrating to the olfactory bulb. Experiments have shown that adult SVZ neurogenesis is mainly involved in mating [108], and in adult paternal recognition of offspring [109]. In order to continue throughout the adulthood, neurogenesis should be tightly regulated to preserve the stem cells pool; otherwise, precursors cells may be depleted earlier by excessive proliferation and differentiation. Finally, evidence shows that adult neurogenesis also occurs in the substantia nigra (SN) in the midbrain at very low numbers in physiological conditions [110]. An increased neuronal turnover is observed after lesions to the SN, indicating that the rate of adult neurogenesis may be regulated in the SN especially after injury [110].

### 4.1. Regulation of Neurogenesis

There are many well-conserved molecular mechanisms that are implicated in regulating neurogenesis within the neurogenic niches in the adult brain. Here, we will briefly present some of the major mechanisms as follows:

#### 4.1.1. Signaling Pathways

There are several pathways involved in maintaining the balance between self-renewal of the precursors and their proliferation and differentiation into mature neurons. One of these pathways is the Notch signaling pathway, which plays an essential role in expanding the neuronal precursor cell pool while keeping them in undifferentiated states [111]. Inactivation of the notch pathway component, recombining binding protein J-kappa (RBPJ), results in an early increased differentiation of the neuronal stem cells and early depletion of the Sox2-positive neuronal precursors, subsequently leading to the suppression of adult neurogenesis [112,113,114]. Another signaling pathway is the Hedgehog pathway, which is activated by the ligand sonic hedgehog (Shh), and is involved in the formation of adult neurogenic niches in the brain [115,116]. Administration of cyclopamine, a pharmacological inhibitor of Shh signaling, in rat brains reduces hippocampal neural progenitor proliferation within the neurogenic niches [115]. Another pathway is the bone morphogenetic proteins (BMPs) signaling pathway, which is activated by ligands accounting for the largest subgroup of the TGFβ superfamily of cytokines. BMPs signaling pathway is highly active in adult hippocampal neurogenesis, triggering several receptor-specific effects including, cell survival, proliferation, and differentiation [90]. Viral-mediated overexpression of BMP4 not only suppresses NSC cell-cycle entry, but also slows down NSC maturation [117]. Furthermore, purified mouse Noggin protein, which is an antagonist to BMP signaling, promotes neurogenesis and neuronal differentiation [118]. Hence, BMP signaling is involved in maintaining the balance between proliferation and quiescence.

Another essential regulatory mechanism is the Wnt signaling, which regroups the canonical pathway, and the non-canonical pathway that comprises Ca^2+^ and planar cell polarity (PCP) signaling pathways. In both pathways, Wnt ligands bind frizzled (Fz) receptors and transduce a signal to the cytoplasmic phosphoprotein Dishevelled (Dvl) [119]. However, the canonical Wnt pathway is β-catenin-dependent, while the non-canonical pathway is β-catenin-independent. Activation of the canonical pathway leads to β-catenin stabilization in the cytosol, mediated by inhibition of glycogen synthase kinase-3β (GSK3β), and its subsequent translocation to the nucleus to regulate the expression of various target genes via the binding to the transcription factor T cell factor/lymphoid enhancer factor (TCF/LEF) [120]. β-catenin signaling was reported to play a key role in regulating the expression of various genes implicated in neurogenesis [121,122]. On the other hand, activation of Wnt/Ca^2+^ pathway increases intracellular Ca^2+^, leading to the activation of protein kinase C (PKC), calcineurin or Ca^2+^/calmodulin-dependent protein kinase-II (CaMKII) intracellular signaling cascades, whereas activation of Wnt/PCP pathway leads to the activation of c-Jun N-terminal kinase (JNK), and Rho-kinase signaling cascades [123]. The activation of Wnt/β-catenin pathway promotes both SGZ precursor proliferation and differentiation, whereas activating the non-canonical Wnt signaling pathways is important for maintaining essentially precursor stemness [93,124]. Wnt3, which is produced by local hippocampal astrocytes, activates Wnt/β-catenin pathway in isolated adult hippocampal precursors and induces their differentiation towards the neuronal lineage [124]. Moreover, activation of the canonical Wnt pathway through TCF/LEF in dividing neuronal progenitors induces the transcription of NeuroD1, which is required for the survival and maturation of adult-born neurons [125,126]. Dickkopf-related protein 1 (Dkk1) and secreted frizzled-related protein 3 (Sfrp3) are both secreted endogenous antagonist of the canonical Wnt pathway [127]. Dkk1 expression increases with age associated to neuronal dysfunction and cognitive decline [128]. Attenuation of Dkk1 expression significantly enhances neurogenesis in the hippocampus [128]. Seib et al. (2013) also demonstrated that neuronal progenitors with inducible loss of Dkk1 developed an increased Wnt activity, resulting in enhanced self-renewal capacity and increased immature neurons generation [128]. Together, these reports highlight the role of several key regulatory pathways that are implicated in controlling of adult neurogenesis.

#### 4.1.2. Epigenetic Regulation

Recent direct evidence suggests that epigenetic regulation, including DNA and histone modifications, as well as noncoding regulatory RNAs, such as microRNA (miRNA) and long noncoding RNA (lncRNA), play an important role in embryonic and adult neurogenesis [129]. DNA methylation is a chemical modification catalyzed by DNA methyltransferases (DNMTs), constituting a major epigenetic factor that regulates cell differentiation. DNMTs mRNA was found to be expressed in all mature neurons in the brain of young and aged mice [130]. Interestingly, there was no significant expression of the gene in the white matter, suggesting a neuron-specific biological function of DNMTs [130]. DNMT1 is expressed in both post-mitotic neurons and dividing neural precursor cells (NPCs) [130]. Prenatal deletion of DNMT1 in NSCs impairs neurogenesis [131], characterized by an abnormal NSCs morphology, migration, and reduced number of integrated neurons in the SGZ of the DG [131]. Moreover, ablation of DNMT1 induces JNK/signal transducers and activators of transcription (JAK/STAT), enhancing glial differentiation, promoting astrogliosis and increasing microglial cell density in the adult mouse brain [132]. On the other hand, DNMT3a is expressed exclusively in post-mitotic neurons [133,134]. Genome wide analysis (GWAS) of post-natal NSCs showed that DNMT3a methylates a large number of transcriptionally permissive genes that regulate neurogenesis [134]. In addition, DNMT3a ablation impairs post-natal neurogenesis in SVZ and SGZ, reducing up to 10 times the number of differentiated neurons in both neurogenic regions [134]. Indeed, conditional knockout of either DNMT1 or DNMT3a in forebrain’s excitatory neurons in mice significantly decreases the volume of DG neurons, compared to controls, suggesting a critical role in neuronal maturation [133]. Interestingly, double knockout animals for DNMT1 and DNMT3a showed no impairments in dividing neurons in the SGZ compared to control, suggesting a role of DNMT1 and DNMT3a in post-mitotic cell maintenance [133]. Moreover, DNMT1 and DNMT3a conditional knockout mice displayed deficits in learning and memory, and showed abnormal long-term plasticity in hippocampal CA1 region [133]. Together, these findings highlight that epigenetic modifications play a critical role in adult neurogenesis regulation. Strong evidence suggests that gene expression and epigenetic modifications are similarly regulated in neurons and microglia (reviewed [135]). For instance, the enzyme Jumonji domain-containing protein D3 (JMJD3) de-methylases H3K27 in the promoter regions of neural lineage genes, thus influencing neuronal differentiation [136]. Similar effects on JMJD3-mediated de-methylation of H3K27 are reported via IL-4 in murine microglial cells [137]. In addition, microglia express spalt-like transcription factor 1 (Sall1) and early growth response protein-1 (Egr1), which are two key transcription factors involved in synaptic pruning and neurogenesis [138]. These evidences indicate that neurons and microglia share common epigenetic modifications associated to key transcription factors (reviewed in [135]). These common changes affect microglia polarization [139], and neurogenesis [140], but further investigations are required to determine the exact mechanisms involved in specifically regulating microglial cell function and neurogenesis via epigenetic modifications.

### 4.2. Physiological Regulation of Neurogenesis by Microglia

Microglia play an important role in maintaining neuronal plasticity [141]. Microglia are majorly found in the neurogenic niches, namely the SVZ and SGZ [142]. Co-culturing microglial cells with the neuropoietic cells, which initially retain its ability to self-renew and then loose it, is essential for neuroblast production [143]. As mentioned earlier, NPCs in SGZ of the DG give rise to newborn neuroblast; however, at the end of 4 weeks, only a small portion of these cells join the hippocampal circuitry as mature neurons. The rest of the newborn cells undergo death by apoptosis, and the apoptotic newborn cells are then cleared through phagocytosis by microglia [76]. Hence, microglia play a key role in shaping the adult hippocampal neurogenesis. Moreover, although the number of newly generated neurons and the apoptotic newborn cells decrease with age and inflammation, the phagocytic feature of microglia remains constant [76]. The role of microglia in neurogenesis is not limited to the clearance and removal of cells, as they are highly involved in the local control of NPCs differentiation to neuroblasts, as well as latter survival, and integration to circuitry (reviewed in [144]). Evidence shows that microglia cells affect the migration and differentiation of NPCs, as precursor cells isolated from the developing brain of mouse migrate toward a gradient of microglia-conditioned media [145]. This observation suggests that microglial cells assist in directing and spatially orienting the migration of NPCs. Aarum et al. (2003) demonstrated that the density of neurons increases in cell cultures treated with microglia-conditioned media, which might be due to the release of microglia-derived soluble factors implicated in the differentiation of precursor cells [145]. The role of microglia in regulating neurogenesis within the SVZ is still a matter of debate. In the adult rodent SVZ, microglia do not express TREM2 [146], suggesting that the phagocytosis phenotype in microglia is lacking in this particular neurogenic region [76,147]. In the olfactory bulb layers, however, microglia express TREM2 and present an amoeboid morphology [147]. Local depletion of microglia in the SVZ via injections of saporin conjugated to CD11b significantly increases the number of neuroblasts in the SVZ, but decreases the number of neuroblasts in the rostral migratory system (RMS) and olfactory bulb [147]. Ribiero Xavier et al. (2015) speculated that the increased number of neuroblasts in SVZ could be due to increased mitotic rate triggered by cytokines in response to the toxic saporin injections, or a result of impaired migration due to the lack of microglial support of newly generated neurons to migrate through the RMS and olfactory bulb [147]. Importantly, the reduced number of neuroblasts in the RMS and olfactory bulb, but not in the SVZ, after microglial cell depletion, suggests that the neuroblasts need microglial support to survive and migrate in the adult neurogenic SVZ region [147]. In contrast, systemic microglial cell ablation using PLX5622 showed no changes after 14 days in neurogenesis nor oligodendrogenesis [148], suggesting that microglia are not required for normal adult SVZ neurogenesis [148]. Nonetheless, Reshef et al. (2017) showed that ablation of microglia with PLX5622 reduces dendritic spine elimination under physiological conditions [149]. Interestingly, depletion of microglia significantly reduces spine density in adult neuroblasts in the SVZ, suggesting that microglia play a critical role in both synapse formation as well as elimination [149]. Finally, evidence shows that phagocytosis of apoptotic bodies in SGZ occurs via unchallenged microglia [7,150], which exhibit a ramified morphology associated to a low expression of inflammatory markers, such as CD11b and CD68 [150].

The association of hippocampal neurogenesis to microglial cell activity was furthered outlined following exposure to external factors, such as environmental enrichment (EE), which has been shown to boost neurogenesis via modulation the functions of microglia and T cells [151]. Interestingly, EE did not enhance adult hippocampal neurogenesis in immune-deficient rats, which was restored by T cells recognizing specific CNS antigens [151]. These findings outline an important role of T-cells in adult neurogenesis by affecting progenitor-cell proliferation. It has been shown that microglial cells cooperate with T helper cells to modulate both neurogenesis and oligodendrogenesis [152]. For instance, it has been shown that the pharmacological suppression of microglial activation results in significant inhibition in neurogenesis and oligodendrogenesis through decreased levels of pro-inflammatory mediators, such as IL1β, IL6, TNFα, and IFNγ [152]. These findings are in line with various reports showing that microglial cell activation enhances neurogenesis [143,145,151] (reviewed in [153]). Such findings demonstrate that microglial polarization and/or the state of NPCs are important factors on the overall outcome on neurogenesis [142,154,155]. Indeed, depending upon the phenotype that microglia adopt, it can either switch towards neurogenesis or oligodendrogenesis. IL4-activated microglial cells promote oligodendrogenesis, whereas IFNγ-activated microglial cells stimulate neurogenesis [156]. Recent evidence shows that phagocytic microglia do not only play a role in passive clearance of apoptotic cells, but also play a central role in controlling adult hippocampal neurogenesis [157]. Chronic depletion of the purinergic receptor P2Y12 and the tyrosine kinases of the TAM family Mer tyrosine kinase (MertK)/Axl in mice impairs adult hippocampal neurogenesis, while the conditional reduced expression of MertK expression transiently increases adult hippocampal neurogenesis [157]. Moreover, Diaz-Aparicio et al. (2020) recently demonstrated that phagocytic microglia provide a negative feedback loop through their secretome to ensure proper proliferation of the newborn cells [157]. Microglial cell implication in maintaining homeostasis in the neurogenic SGZ niche via their secretome is in line with recent work highlighting the role of macrophages in regulating stem cell niches (reviewed in [158]). Furthermore, involvement of the microglial purinergic receptor P2Y12 in modulating neurogenesis was highlighted in epilepsy, which has been shown to promoting aberrant adult hippocampal neurogenesis by increasing immature neuronal projections associated to seizures [159]. Neuronal-microglial crosstalk via the CX3CL1-CX3CR1 pathway has also been implicated in regulating adult neurogenesis (reviewed in [144]). CX3CR1 knockout mice display decreased hippocampal neurogenesis, and mediated essentially by increased levels of IL1 [160]. In addition, CX3CR1 knockout mice have smaller spine heads in adult born NPCs in the SVZ [149]. Decreased spine size has been previously correlated with synaptic efficacy [161]. These reports indicate that the CX3CL1-CX3CR1 pathway plays an essential role in regulating synaptic development as well as the generation of newborn neurons in the adult brain.

There is direct evidence suggesting that microglial cell activity and neurogenesis in the adult hippocampus are influenced by physiological conditions such as aging and exercise [162]. Indeed, voluntary exercise induces neurogenesis in the DG of adult mice [163,164]. In the context of microglial control of neurogenesis associated to physical exercise, the findings suggest that the exercise-induced increase in hippocampal neurogenesis is associated with CX3CL1-CX3CR1 pathway [162]. Vukovic et al. (2012) have demonstrated that the exercise-induced increase in hippocampal neurogenesis is associated with a decreased number of major histocompatibility complex class II (MHCII)-positive microglia [162]. These findings suggest that microglial cell activity, under normal physiological conditions and exercise, regulates adult hippocampal neurogenesis. The age-dependent decrease in neurogenesis is associated to an age-related upregulation of the BMPs signaling pathway [165,166]. Moreover, Dkk1, a canonical Wnt pathway antagonist, increases with age in the adult hippocampus and inhibits SGZ precursors proliferation and differentiation [128]. Loss of Dkk1 enhances neurogenesis and counteracts the age-related cognitive decline [124,128]. The age-related decrease in neurogenesis and the associated cognitive decline can be attenuated by EE. Indeed, EE was found to improve cognition in several animal models of neurodegenerative diseases [167]. Specifically, EE increases the proliferation and survival of newborn adult neurons in the SGZ of the DG [168]. Moreover, exercise-induced neurogenesis has been shown to delay the age-related cognitive decline [169]. Exercise using a wheel-runner was found to reverse neurogenesis decline in aged mice compared to controls [170]. Interestingly, running reduces the age-associated morphological deficits of newborn adult neurons [170]. In parallel, treadmill running attenuates the age-dependent increase in aberrant microglia activation in a transgenic model of Alzheimer’s disease (AD) [171]. Running was also found to reduce the number of hyper-activated microglia in the brain of aged mice and increase their pro-neurogenic phenotype by increasing the expression of growth factors, namely insulin-like growth factors (IGF1) [172]. Indeed, IGF-1 release by microglia was reported after EE [151]. These findings suggest that physical exercise and EE may enhance neurogenesis by modulating microglial cell activity/activation and prompting their transition towards a proneurogenic phenotype. Finally, microglia-derived TGFβ was also shown to regulate neurogenesis (reviewed in [173]), by enhancing neuronal differentiation and neuronal survival [174,175], and reducing the pool of NSCs in the hippocampus [176]. These reports demonstrate that microglial cells play a complex yet critical role in modulating adult neurogenesis, but more investigations are still required to fill-in the gaps in the literature.

### 4.3. Pathological Regulation of Neurogenesis by Microglia

Under pathological conditions, microglia change their morphology by adopting an amoeboid-like phenotype, and acquire activation-specific phenotypes, such as phagocytosis, and secrete a various range of inflammatory mediators [4,177]. In addition to the previously mentioned physiological contribution of microglia in regulating neurogenesis, there is evidence that an excessive pro-inflammatory phenotype associated to pathological stimuli may also have profound consequences on neurogenesis. For example, intraperitoneal injection of lipopolysaccharide (LPS) in adult rats impairs neurogenesis by stimulating a pro-inflammatory response in microglia within the neurogenic niches, characterized by IL1β, IL6, and TNFα release [178]. Ekdahl et al. (2003) also found that the number of the surviving newborn neurons in the hippocampus negatively correlates with the number of activated microglia. Indeed, induction of chronic inflammation using cranial radiation increases the number of activated microglia in the brain of rats, and impairs adult hippocampal neurogenesis [179]. Exposure of progenitor cells in vitro to recombinant IL6 significantly decreases neurogenesis, while administration of IL1β, or IFNγ did not [179]. Nonetheless, recent findings demonstrate that the pro-inflammatory phenotype of microglia has no effect on the survival of NPCs, indicating that more investigations are needed to fully understand the role of neuroinflammation on adult neurogenesis [157]. Furthermore, chronic neuropathic pain induced by the chronic construction injury (CCI) of the sciatic nerve in C57BL/6 aged mice was shown to cause memory impairments [180]. Neuropathic pain stimulates the transition of activated microglial state towards a pro-inflammatory phenotype, associated to reduced adult hippocampal neurogenesis [180]. Moreover, evidence shows that the vacuolar sorting protein 35 (VPS35) has an essential role in the pathological microglial regulation of neurogenesis. This protein is a component of retromer, which has an essential role in recycling cargo molecules from endosomes to the trans-Golgi network [181]. VPS35 regulates microglial activity, and VPS35 is significantly decreased in AD patients [182]. Mice lacking VPS35 have impaired hippocampal neurogenesis associated to an increased NPCs proliferation without normal differentiation [183]. VPS35-depleted mice exhibited impaired long-term memory and exacerbated microglial phagocytic activity when assessed in vitro [183]. Together, these findings indicate that microglial VSP35 is a key regulator of adult hippocampal neurogenesis and that its downregulation disrupts hippocampal homeostasis. Additionally, intrastriatal thrombin injection induces microglial activation resulting in impaired adult hippocampal neurogenesis [184]. Conversely, administration of a direct thrombin inhibitor, indometacin, reduces microglial excessive activation, enhances neurogenesis, and subsequently improves spatial memory in mice [184]. Taken together, these reports outline a key role activated microglia associated to pathological neuroinflammatory conditions impair adult neurogenesis.

## 5. Microglia-Mediated Regulation of Neurogenesis in Aging and Neurodegeneration

Aging is characterized by a progressive decline in the physiological and biological functions of all organs and cells, resulting in an increased susceptibility to disease and death. Many studies show that neurogenesis decreases with age [185,186,187] (reviewed in [188]). Recently, inconsistent findings were reported in humans regarding neurogenesis during aging (reviewed in [189]); Sorrells et al. (2018) reported a significant age-dependent decrease in adult hippocampal neurogenesis in postmortem samples [190], while others showed that adult hippocampal neurogenesis was relatively preserved [191]. During aging, microglia become less mobile [192], chronically express pro-inflammatory cytokines [193], and their phagocytic capacity gets impaired [194,195]. It is well established that inflammation is associated with normal aging as well as neurodegenerative diseases; potentially influencing adult neurogenesis. Microglia can either adopt a neuroprotective or neuroinflammatory phenotype, depending upon the biological context [196]. The process of aging progressively shifts the brain towards mild neuroinflammatory state and alters microglial activity that adopt a pro-inflammatory neurodestructive phenotype [197]. Indeed, aged microglia express higher levels of pro-inflammatory cytokines compared to their young counterparts [193]. The pro-inflammatory microglial phenotype is accompanied by the release of various neurotoxic cytokines such as IL1β, IL16, and TNFα, which have been shown to impair neurogenesis [172,198,199]. CX3CR1 knockout mice have decreased NPCs proliferation and a decreased number of neuroblasts [200]. Indeed, a disrupted CX3CL1/CX3CR1 axis (microglial-neuron communication) is associated with increased IL1β protein levels, and the blockage of IL1-R1 was sufficient to reverse long-term potentiation (LTP) and cognitive impairments in CX3CR1 knockout mice [200]. Importantly, the release of CX3CL1 is reduced in aged brains [162]. Hence, altered CX3CL1/CX3CR1 signaling during aging could underlie neurogenesis impairments (reviewed in [201]). In this regard, CX3CR1 depletion in animal models of AD significantly reduces AD-related pathology by enhancing microglial phagocytic ability [202,203,204].

Aging is a known risk factor for several neurological conditions, including AD and Parkinson’s disease (PD). AD is an age-related, progressive and irreversible neurodegenerative disorder characterized by extracellular deposition of beta-amyloid (Aβ) plaques, intracellular deposition of neurofibrillary tangles, and neuronal death [205]. The sustained inflammatory response triggered by Aβ brain accumulation might be responsible for the tauopathy, synaptic toxicity and dysfunction, and consequently neuronal death [206,207]. The hippocampus is affected early in AD, and recently it has been proposed that impaired adult neurogenesis constitutes an early event in AD caused by the intracellular Aβ oligomers [208]. However, there has been conflicting reports about whether neurogenesis is enhanced or repressed in AD, and is still a matter of debate that requires further clarification [209]. Nonetheless, recent evidence from human hippocampal post-mortem tissue demonstrates a clear decrease in the number of NPCs and neuroblasts that even preceded tangle and plaque formation in individuals with mild cognitive impairment [210]. In line with these findings, Tobin et al. (2019) have demonstrated reduced number of neuroblasts in individuals with mild cognitive impairment, correlating with a higher number of neuroblasts and better cognitive functions and synaptic strength [211]. Moreover, some mutations associated to elevated risk of AD must be taken into account as they may affect neurogenesis differently. For example, NPCs-derived from mice expressing the presinilin-1 (PS1) variant associated to familial AD show no difference in proliferation and differentiation when compared to wild-type controls [212]. However, when the same NPCs were co-cultured with PS1-expressing microglia, a significant decrease was observed in proliferation and neuronal differentiation [212]. This suggests that microglia may play a central role in PS1-mediated deficits in adult hippocampal neurogenesis. Moreover, evidence shows that the familial PS1 A246E mutation stabilizes β-catenin, which is involved in Wnt pathway, resulting in an increased proliferation of NPCs in the DG of adult mice without affecting their survival or differentiation [213]. Conversely, experiments in transgenic mice having the mutated form of the amyloid precursor protein (APP) showed decreased proliferation and survival of NPCs in the DG of the hippocampus [214]. Interestingly, Jin et al. (2004) detected 2-fold enhancement in neurogenesis in both the DG and SVZ in AD transgenic (PDGF-APPSw, Ind) mice, which express the Swedish and Indiana APP mutations [215]. These findings were observed before the detection of neuronal loss and Aβ deposition. Similarly, an increase in neurogenesis was detected in the brain of AD patients using Dcx, which is a microtubule-associated neuronal protein, as well as other neurogenesis markers [216].

On the other hand, homozygous PDAPP mice, which overexpress the human APP V717F mutation, show an age-dependent decrease in precursor proliferation within the SGZ [217]. However, 1-year old PDAPP mice had increased rate of immature neurons in the outer portion of the granule cell layer, potentially explaining why some reports linked AD with increased neurogenesis [217]. Using humans NPCs, Haughey et al. (2002) showed that Aβ oligomers impairs the proliferation and neuronal differentiation of the NPCs and induces their apoptosis by deregulating the cellular Ca^2+^ homeostasis and the activation of calpains and caspases [214]. Furthermore, an enhanced neurogenesis was observed within the SVZ in vivo or in vitro after administration of Aβ_1–42_ in the SVZ of young adult mice [218]. This increase in neurogenesis was associated with high expression levels of p75 neurotrophin cell receptor (p75^NTR^) [218]. Surprisingly, precursor cells from an old APP/PS1 mice failed to respond to Aβ_1–42_, suggesting that overstimulation of p75 receptor early in life may deplete the NSC pool and impair neurogenesis later in life [218]. A decrease in SGZ neurogenesis was also reported using HH3, a proliferating mitotic marker, in 3xTg-AD mice, which harbor 3 mutant genes for APPswe, for PS1m146, and for taup301L. This decrease in adult neurogenesis in the SGZ worsens with age and was directly associated with Aβ plaques [219]. Similarly, decreased adult neurogenesis in the SGZ and SVZ of 3xTg-AD mice was reported early in the disease before the development Aβ plaques and neurofibrillary tangles [220]. It is noteworthy to mention that evidence has also linked microglia to a role in facilitating AD pathology. For example, the expression of IGF1, which is implicated in regulating neurogenesis, significantly increases in APP/PS1 mice along with an increased activation of microglia, and a reduction in neurogenesis in the SGZ [221]. Furthermore, the reduction of TGFβ in another AD mouse model led to accelerated neurodegeneration and AD-like pathology [222]. Together, these findings point to IGF1, and TGFβ as a potential target in modulating adult hippocampal neurogenesis in AD. Moreover, Biscaro et al. (2012) found that administration of minocycline, a tetracycline derivative, reduced the number and activity of microglial cells interacting with Aβ deposits in the DG of APP/PS1 mice [199]. Minocycline also normalizes the increased hippocampal level of IL6 and TNFα in APP/PS1 mice, and increases the survival of the newly born neurons [199]. Interestingly, minocycline had no effect on cell proliferation and differentiation as indicated by the numbers of Ki67+ cells, and both brain levels of Aβ and Aβ-related morphological deficits remained unaffected by minocycline [199]. Hence, in APP/PS1 mice, decreased microglial activity could be beneficial for adult hippocampal neurogenesis. Aβ peptide is known to cause abnormal activation of cyclin-dependent kinase 5 (CDK5) enzyme leading to tau hyper-phosphorylation (Reviewed in [223]). Using APPswe/PS1E9 mice, which express a chimeric mouse/human APP (Mo/HuAPP695swe) and mutant human PS1-dE9, an increase in the levels of Aβ and phosphorylated tau was reported in the neurogenic niches and was demonstrated to impair neurogenesis [224]. Other findings indicate that APPswe/PS1E9 mice display a significantly higher number of activated microglia in the DG in comparison to controls [225]. Indeed, evidence demonstrates that microglia play a central role in the spreading of tau and synaptic dysfunction in AD (reviewed in [226]). Additionally, several experimental findings using different animal models of tauopathy suggest that tau phosphorylation is sufficient to trigger microglial activation and promote neuroinflammation [227,228,229,230]. However, the exact mechanisms underlying how the microglial-control of neurogenesis is influenced by pathological hallmarks of AD remain elusive. In summary, these findings illustrate that distinct mechanisms could underlie the regulation of adult neurogenesis in AD, and that further studies are needed to elucidate their exact role in the pathogenesis of AD (Table 1).

The neuropathological cascade in PD includes the selective degeneration of dopaminergic neurons in the SN, and the deposition of cytoplasmic Lewy bodies, which is composed of ubiquitin and α-synuclein, and gliosis [238]. Impairments in adult neurogenesis have been reported in PD patients [233,234,239], as well as in transgenic animal models of PD [231,233,240,241,242] (reviewed in [243]). In vivo, 1-methyl-4-phenyl-1,2,3,6-tetrahydropyridine (MPTP) and 6-hydroxydopamine (6-OHDA) mediated lesion models of PD with loss of dopaminergic nigrostriatal tract show a robust decrease in NPCs proliferation at the SVZ [232,234,244,245]. Interestingly, neurogenesis reduction that is observed in the SVZ positively correlates with the severity of dopaminergic denervation [244]. Administration of Levodopa, a dopamine precursor, to dopamine-depleted animals was sufficient to recover NPCs proliferation in the SVZ, suggesting that dopaminergic input is necessary for neurogenesis in the SVZ [234]. In addition, microgliosis has been reported in the olfactory bulb in PD patients [246] and mouse models [247]. Potential mechanisms underlying the control of neurogenesis in the SVZ by dopaminergic neurons include interactions between dopamine and the epidermal growth factor (EGF) [239], and the ciliary neurotrophic factor (CNTF) [248]. Importantly, recent evidence shows that microglia express the CNTF receptor alpha (CNTFα), and that CNTF inhibits microglial pro-inflammatory activation and microglial-derived oxidative stress in a PD mouse model [249]. Hence, the CNTF pathway in microglia could be a potential mechanism through which neurogenesis in the SVZ is affected in PD. Indeed, it is well established that neuroinflammation plays a central role in the pathogenesis of PD [250]. Similarly to what has been reported in AD, the advanced stages of PD are associated with severe hippocampal atrophy, which may partly explain why neurogenesis in the SGZ is substantially decreased in PD [232,233] (reviewed in [243]). Nonetheless, several other reports showed either no effects or an increased effect of dopaminergic depletion in animals on SGZ and SVZ neurogenesis [235,236], suggesting that non-dopaminergic changes in PD may underlie the impairments in neurogenesis. Dopaminergic depletion models of PD is associated with neuroinflammation, thus providing a framework to better characterize the exact contribution of microglial cell activity/activation as well as other key pathways such as the canonical Wnt/β catenin, to the impaired neurogenesis (reviewed in [251]). For example, evidence shows that the impaired neurogenesis at the SVZ following MPTP injection is partly due to an exacerbated pro-inflammatory response triggered by activated microglia associated to upregulation of the phagocyte oxidase, and downregulation of the Wnt/β catenin pathway [237,252]. Indeed, recent data demonstrate that D1 receptors regulate dopaminergic neurogenesis in the SN and mitochondrial functions via stimulating the Wnt/β catenin pathway in 6-OHDA rat model of PD [253]. These findings outline the importance of the crosstalk between microglia and the Wnt/β catenin pathway in maintaining homeostasis in the SVZ neurogenic niche, and dopaminergic neurogenesis in the SN, in the context of PD. Whether these mechanisms and pathways also contribute to the regulation of adult neurogenesis in the SGZ in PD models remains unclear. Together, evidence suggests that adult neurogenesis is impaired in PD; however, the exact role of microglia in regulating neurogenesis in PD remains to be elucidated.

## 6. Concluding Remarks

**Figure 1:**
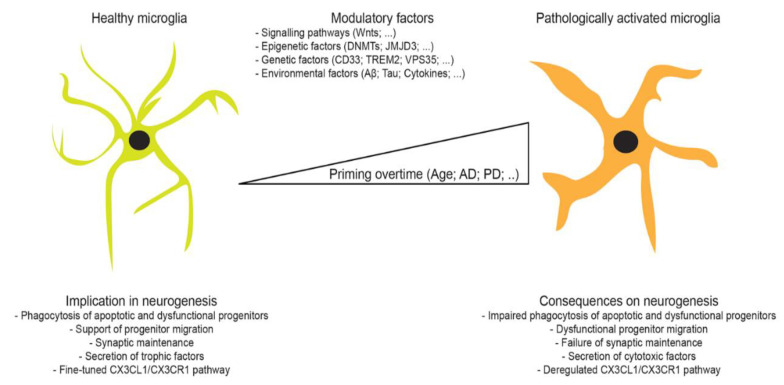
Microglia regulation of neurogenesis during lifespan: In the young healthy brain, microglia actively modulate neurogenesis via several supportive mechanisms that ensure the proper turnover of adult newly born neurons. A plethora of modulatory factors dictate microglial functions, and thereby influencing the rate of adult neurogenesis. Over age, microglia become less mobile, adopt an amoeboid-like phenotype, chronically express pro-inflammatory cytokines, and display an impaired phagocytic activity. The age-dependent establishment of a pro-inflammatory phenotype is accompanied by the release of neurotoxic cytokines, which impairs neurogenesis and synaptic integrity, and thereby contributing to neuronal loss and cognitive decline seen in Alzheimer’s disease (AD), and Parkinson’s disease (PD).

Under physiological conditions, microglia exhibit a morphology characterized by dense long processes, which continuously scan their microenvironment to sense the presence of abnormal signals. Microglia rapidly respond once a signal is detected by orchestrating specific responses, which include the release of cytokines and chemokines, and phagocytosis, in order preserve brain structural and functional integrity at the biochemical and cellular level. A large body of evidence points to a central role of microglial cell function in the biology of aging and neurodegenerative disease (Figure 1). The overwhelming findings suggest that microglia attempt to prevent AD pathogenesis via Aβ elimination at early stages, but largely fail on the long-term as the pathogenesis progresses, notably due to the excessive accumulation of Aβ and adoption of a pro-inflammatory phenotype (Figure 1). Similarly, microglia are implicated in regulating adult neurogenesis, a biological process that helps in promoting neuronal function. While further investigations are needed to determine how neurogenesis changes with age in humans, the majority of the experimental findings in various animal models clearly indicate that neurogenesis is impaired in an age-dependent manner (Table 1) (reviewed in [254]). The recent findings are highlighting an unexpectedly complex, yet fascinating, role of microglia in regulating adult neurogenesis. However, further evidence is still required to fully address the mechanism mechanisms underlying microglial cell contribution in modulating neurogenesis in aging and neurodegenerative diseases, and to characterize the subsequent outcomes. A better understanding of the regulatory mechanisms that trigger microglial dysfunction during age and neurodegenerative diseases, including AD and PD, would allow the development of novel immunomodulatory therapeutic interventions that aim essentially to influence neurogenesis via modulation of microglial cell responses (reviewed in [255]).

## Figures and Tables

**Table 1:** Summary of models of neurodegeneration and the reported effects on neurogenesis (NPC: neural precursor cell; PS1: presinilin-1; SGZ: subgranular zone; SVZ: subventricular zone; DG: dentate gyrus; AD: Alzheimer’s Disease; PD: Parkinson’s Disease; 6-OHDA: 6-hydroxydopamine; MPTP: 1-methyl-4-phenyl-1,2,3,6-tetrahydropyridine).

Model of Neurodegeneration	Effect on Neurogenesis
NPCs from mice expressing the PS1 variant co-cultured with PS1-expressing microglia [212]	Decreased proliferation and differentiation
Mice with familial PS1 A246E mutation [213]	Increased proliferation of NPCs in the SGZ, no effect on survival and differentiation
Mice with mutated form of APP [214]	Decreased proliferation and survival of NPCs in the DG
Mice with PDGF-APPSw,Ind mutation [215]	Two fold enhancement in proliferation in both the DG and SVZ
AD human patients [216]	Increased proliferation and differentiation in the DG
AD human patients [210]	Decreased proliferation and survival in the DG
Mice with PDAPP mutation [217]	Age-dependent decrease in proliferation in the SGZ
Administration of Aβ_1–42_ in vitro [218]	Increased proliferation in the SVZ
3xTg-AD mice [219,220]	Age-dependent decrease in neurogenesis in the SGZ
APP/PS1 mice [224]	Decreased neurogenesis in the SGZ
APP/PS1 mice treated with minocycline [199]	Increased survival in the SGZ, no effect on cell proliferation and differentiation
Overexpression of human wild-type α-synuclein model of PD in mice [231]	Decreased survival in the SGZ
MPTP model of PD in mice [232]	Decreased proliferation in the SVZ
6-OHDA model of PD in rats [232]	Decreased proliferation in the SVZ
PD human patients [233]	Decreased proliferation in the SGZ
PD human patients [234]	Decreased proliferation in the SVZ
MPTP model of PD in mice [235]	Transient increase in proliferation in the SGZ
6-OHDA model of PD in rats [236]	No effect on proliferation or differentiation in the SGZ
MPTP model of PD in mice [237]	Decreased proliferation in the SVZ

## References

[1] ElAli A., Rivest S. (2016). Microglia Ontology and Signaling. Front. Cell Dev. Biol..

[2] Ginhoux F., Prinz M. (2015). Origin of microglia: Current concepts and past controversies. Cold Spring Harb. Perspect. Biol..

[3] Chan W.Y., Kohsaka S., Rezaie P. (2007). The origin and cell lineage of microglia: New concepts. Brain Res. Rev..

[4] Kettenmann H., Hanisch U.K., Noda M., Verkhratsky A. (2011). Physiology of microglia. Physiol. Rev..

[5] Kettenmann H., Kirchhoff F., Verkhratsky A. (2013). Microglia: New roles for the synaptic stripper. Neuron.

[6] Lampron A., Elali A., Rivest S. (2013). Innate immunity in the CNS: Redefining the relationship between the CNS and Its environment. Neuron.

[7] Davalos D., Grutzendler J., Yang G., Kim J.V., Zuo Y., Jung S., Littman D.R., Dustin M.L., Gan W.B. (2005). ATP mediates rapid microglial response to local brain injury in vivo. Nat. Neurosci..

[8] Nimmerjahn A., Kirchhoff F., Helmchen F. (2005). Resting microglial cells are highly dynamic surveillants of brain parenchyma in vivo. Science.

[9] Kumar H., Kawai T., Akira S. (2011). Pathogen recognition by the innate immune system. Int. Rev. Immunol..

[10] Nadeau S., Rivest S. (2000). Role of microglial-derived tumor necrosis factor in mediating CD14 transcription and nuclear factor kappa B activity in the brain during endotoxemia. J. Neurosci..

[11] Kuno R., Wang J., Kawanokuchi J., Takeuchi H., Mizuno T., Suzumura A. (2005). Autocrine activation of microglia by tumor necrosis factor-alpha. J. Neuroimmunol..

[12] Feng X.H., Derynck R. (2005). Specificity and versatility in tgf-beta signaling through Smads. Annu. Rev. Cell Dev. Biol..

[13] Suzumura A., Sawada M., Yamamoto H., Marunouchi T. (1993). Transforming growth factor-beta suppresses activation and proliferation of microglia in vitro. J. Immunol..

[14] Letterio J.J., Roberts A.B. (1998). Regulation of immune responses by TGF-beta. Annu. Rev. Immunol..

[15] Ledeboer A., Breve J.J., Poole S., Tilders F.J., Van Dam A.M. (2000). Interleukin-10, interleukin-4, and transforming growth factor-beta differentially regulate lipopolysaccharide-induced production of pro-inflammatory cytokines and nitric oxide in co-cultures of rat astroglial and microglial cells. Glia.

[16] Fernandez E.J., Lolis E. (2002). Structure, function, and inhibition of chemokines. Annu. Rev. Pharmacol. Toxicol..

[17] Sokol C.L., Luster A.D. (2015). The chemokine system in innate immunity. Cold Spring Harb. Perspect. Biol..

[18] Paolicelli R.C., Bisht K., Tremblay M.E. (2014). Fractalkine regulation of microglial physiology and consequences on the brain and behavior. Front. Cell Neurosci..

[19] El Khoury J., Toft M., Hickman S.E., Means T.K., Terada K., Geula C., Luster A.D. (2007). Ccr2 deficiency impairs microglial accumulation and accelerates progression of Alzheimer-like disease. Nat. Med..

[20] Liu H., Leak R.K., Hu X. (2016). Neurotransmitter receptors on microglia. Stroke Vasc. Neurol..

[21] Inoue K. (2002). Microglial activation by purines and pyrimidines. Glia.

[22] Burnstock G., Verkhratsky A. (2010). Long-term (trophic) purinergic signalling: Purinoceptors control cell proliferation, differentiation and death. Cell Death Dis..

[23] Illes P., Alexandre Ribeiro J. (2004). Molecular physiology of P2 receptors in the central nervous system. Eur. J. Pharmacol..

[24] Farber K., Markworth S., Pannasch U., Nolte C., Prinz V., Kronenberg G., Gertz K., Endres M., Bechmann I., Enjyoji K. (2008). The ectonucleotidase cd39/ENTPDase1 modulates purinergic-mediated microglial migration. Glia.

[25] Xiang Z., Chen M., Ping J., Dunn P., Lv J., Jiao B., Burnstock G. (2006). Microglial morphology and its transformation after challenge by extracellular ATP in vitro. J. Neurosci. Res..

[26] Lee M. (2013). Neurotransmitters and microglial-mediated neuroinflammation. Curr. Protein. Pept. Sci..

[27] Noda M., Nakanishi H., Nabekura J., Akaike N. (2000). AMPA-kainate subtypes of glutamate receptor in rat cerebral microglia. J. Neurosci..

[28] Kaushal V., Schlichter L.C. (2008). Mechanisms of microglia-mediated neurotoxicity in a new model of the stroke penumbra. J. Neurosci..

[29] Kuhn S.A., van Landeghem F.K., Zacharias R., Farber K., Rappert A., Pavlovic S., Hoffmann A., Nolte C., Kettenmann H. (2004). Microglia express GABA(B) receptors to modulate interleukin release. Mol. Cell Neurosci..

[30] Mead E.L., Mosley A., Eaton S., Dobson L., Heales S.J., Pocock J.M. (2012). Microglial neurotransmitter receptors trigger superoxide production in microglia; consequences for microglial-neuronal interactions. J. Neurochem..

[31] Lee M., Schwab C., McGeer P.L. (2011). Astrocytes are GABAergic cells that modulate microglial activity. Glia.

[32] De Simone R., Ajmone-Cat M.A., Carnevale D., Minghetti L. (2005). Activation of alpha7 nicotinic acetylcholine receptor by nicotine selectively up-regulates cyclooxygenase-2 and prostaglandin E2 in rat microglial cultures. J. Neuroinflamm..

[33] Shytle R.D., Mori T., Townsend K., Vendrame M., Sun N., Zeng J., Ehrhart J., Silver A.A., Sanberg P.R., Tan J. (2004). Cholinergic modulation of microglial activation by alpha 7 nicotinic receptors. J. Neurochem..

[34] O’Donnell J., Zeppenfeld D., McConnell E., Pena S., Nedergaard M. (2012). Norepinephrine: A neuromodulator that boosts the function of multiple cell types to optimize CNS performance. Neurochem. Res..

[35] Zhang Y., Chen K., Sloan S.A., Bennett M.L., Scholze A.R., O’Keeffe S., Phatnani H.P., Guarnieri P., Caneda C., Ruderisch N. (2014). An RNA-sequencing transcriptome and splicing database of glia, neurons, and vascular cells of the cerebral cortex. J. Neurosci..

[36] Dello Russo C., Boullerne A.I., Gavrilyuk V., Feinstein D.L. (2004). Inhibition of microglial inflammatory responses by norepinephrine: Effects on nitric oxide and interleukin-1beta production. J. Neuroinflamm..

[37] Nakamura Y. (2002). Regulating factors for microglial activation. Biol. Pharm. Bull..

[38] Mori K., Ozaki E., Zhang B., Yang L., Yokoyama A., Takeda I., Maeda N., Sakanaka M., Tanaka J. (2002). Effects of norepinephrine on rat cultured microglial cells that express alpha1, alpha2, beta1 and beta2 adrenergic receptors. Neuropharmacology.

[39] Farber K., Pannasch U., Kettenmann H. (2005). Dopamine and noradrenaline control distinct functions in rodent microglial cells. Mol. Cell Neurosci..

[40] Bisogno T., Di Marzo V. (2010). Cannabinoid receptors and endocannabinoids: Role in neuroinflammatory and neurodegenerative disorders. CNS Neurol. Disord. Drug Targets.

[41] Colonna M. (2003). TREMs in the immune system and beyond. Nat. Rev. Immunol..

[42] Fu R., Shen Q., Xu P., Luo J.J., Tang Y. (2014). Phagocytosis of microglia in the central nervous system diseases. Mol. Neurobiol..

[43] Wang Y., Cella M., Mallinson K., Ulrich J.D., Young K.L., Robinette M.L., Gilfillan S., Krishnan G.M., Sudhakar S., Zinselmeyer B.H. (2015). TREM2 lipid sensing sustains the microglial response in an Alzheimer’s disease model. Cell.

[44] Stefano L., Racchetti G., Bianco F., Passini N., Gupta R.S., Panina Bordignon P., Meldolesi J. (2009). The surface-exposed chaperone, Hsp60, is an agonist of the microglial TREM2 receptor. J. Neurochem..

[45] Martin S.J., Reutelingsperger C.P., McGahon A.J., Rader J.A., van Schie R.C., LaFace D.M., Green D.R. (1995). Early redistribution of plasma membrane phosphatidylserine is a general feature of apoptosis regardless of the initiating stimulus: Inhibition by overexpression of Bcl-2 and Abl. J. Exp. Med..

[46] Ravichandran K.S. (2011). Beginnings of a good apoptotic meal: The find-me and eat-me signaling pathways. Immunity.

[47] De S.R., Ajmone-Cat M.A., Nicolini A., Minghetti L. (2002). Expression of phosphatidylserine receptor and down-regulation of pro-inflammatory molecule production by its natural ligand in rat microglial cultures. J. Neuropathol. Exp. Neurol..

[48] Canton J., Neculai D., Grinstein S. (2013). Scavenger receptors in homeostasis and immunity. Nat. Rev. Immunol..

[49] de Winther M.P., van Dijk K.W., Havekes L.M., Hofker M.H. (2000). Macrophage scavenger receptor class A: A multifunctional receptor in atherosclerosis. Arterioscler. Thromb. Vasc. Biol..

[50] Coller S.P., Paulnock D.M. (2001). Signaling pathways initiated in macrophages after engagement of type A scavenger receptors. J. Leukoc. Biol..

[51] Granucci F., Petralia F., Urbano M., Citterio S., Di Tota F., Santambrogio L., Ricciardi-Castagnoli P. (2003). The scavenger receptor MARCO mediates cytoskeleton rearrangements in dendritic cells and microglia. Blood.

[52] Coraci I.S., Husemann J., Berman J.W., Hulette C., Dufour J.H., Campanella G.K., Luster A.D., Silverstein S.C., El-Khoury J.B. (2002). CD36, a class B scavenger receptor, is expressed on microglia in Alzheimer’s disease brains and can mediate production of reactive oxygen species in response to beta-amyloid fibrils. Am. J. Pathol..

[53] Febbraio M., Hajjar D.P., Silverstein R.L. (2001). CD36: A class B scavenger receptor involved in angiogenesis, atherosclerosis, inflammation, and lipid metabolism. J. Clin. Investig..

[54] El Khoury J.B., Moore K.J., Means T.K., Leung J., Terada K., Toft M., Freeman M.W., Luster A.D. (2003). CD36 mediates the innate host response to beta-amyloid. J. Exp. Med..

[55] Kobayashi K., Imagama S., Ohgomori T., Hirano K., Uchimura K., Sakamoto K., Hirakawa A., Takeuchi H., Suzumura A., Ishiguro N. (2013). Minocycline selectively inhibits M1 polarization of microglia. Cell Death Dis..

[56] Perego C., Fumagalli S., De Simoni M.G. (2011). Temporal pattern of expression and colocalization of microglia/macrophage phenotype markers following brain ischemic injury in mice. J. Neuroinflamm..

[57] Reiss A.B., Anwar K., Wirkowski P. (2009). Lectin-like oxidized low density lipoprotein receptor 1 (LOX-1) in atherogenesis: A brief review. Curr. Med. Chem..

[58] Zhang D., Sun L., Zhu H., Wang L., Wu W., Xie J., Gu J. (2012). Microglial LOX-1 reacts with extracellular HSP60 to bridge neuroinflammation and neurotoxicity. Neurochem. Int..

[59] Crocker P.R., Paulson J.C., Varki A. (2007). Siglecs and their roles in the immune system. Nat. Rev. Immunol..

[60] Daeron M. (1997). Fc receptor biology. Annu. Rev. Immunol..

[61] Griciuc A., Serrano-Pozo A., Parrado A.R., Lesinski A.N., Asselin C.N., Mullin K., Hooli B., Choi S.H., Hyman B.T., Tanzi R.E. (2013). Alzheimer’s disease risk gene CD33 inhibits microglial uptake of amyloid beta. Neuron.

[62] Claude J., Linnartz-Gerlach B., Kudin A.P., Kunz W.S., Neumann H. (2013). Microglial CD33-related Siglec-E inhibits neurotoxicity by preventing the phagocytosis-associated oxidative burst. J. Neurosci..

[63] Crehan H., Holton P., Wray S., Pocock J., Guerreiro R., Hardy J. (2012). Complement receptor 1 (CR1) and Alzheimer’s disease. Immunobiology.

[64] Crehan H., Hardy J., Pocock J. (2013). Blockage of CR1 prevents activation of rodent microglia. Neurobiol. Dis..

[65] Hall A.A., Herrera Y., Ajmo C.T., Cuevas J., Pennypacker K.R. (2009). Sigma receptors suppress multiple aspects of microglial activation. Glia.

[66] Wu Z., Li L., Zheng L.T., Xu Z., Guo L., Zhen X. (2015). Allosteric modulation of sigma-1 receptors by SKF83959 inhibits microglia-mediated inflammation. J. Neurochem..

[67] Heiss K., Vanella L., Murabito P., Prezzavento O., Marrazzo A., Castruccio Castracani C., Barbagallo I., Zappala A., Arena E., Astuto M. (2016). (+)-Pentazocine reduces oxidative stress and apoptosis in microglia following hypoxia/reoxygenation injury. Neurosci. Lett..

[68] Zhao J., Ha Y., Liou G.I., Gonsalvez G.B., Smith S.B., Bollinger K.E. (2014). Sigma receptor ligand, (+)-pentazocine, suppresses inflammatory responses of retinal microglia. Investig. Ophthalmol. Vis. Sci..

[69] Xu Y., He H., Li C., Shi Y., Wang Q., Li W., Song W. (2011). Immunosuppressive effect of progesterone on dendritic cells in mice. J. Reprod. Immunol..

[70] Bali N., Morgan T.E., Finch C.E. (2013). Pgrmc1: New roles in the microglial mediation of progesterone-antagonism of estradiol-dependent neurite sprouting and in microglial activation. Front. Neurosci..

[71] Hoek R.M., Ruuls S.R., Murphy C.A., Wright G.J., Goddard R., Zurawski S.M., Blom B., Homola M.E., Streit W.J., Brown M.H. (2000). Down-regulation of the macrophage lineage through interaction with OX2 (CD200). Science.

[72] Schmidt A.M., Yan S.D., Yan S.F., Stern D.M. (2001). The multiligand receptor RAGE as a progression factor amplifying immune and inflammatory responses. J. Clin. Investig..

[73] Tremblay M.E., Stevens B., Sierra A., Wake H., Bessis A., Nimmerjahn A. (2011). The role of microglia in the healthy brain. J. Neurosci..

[74] Ransohoff R.M., Perry V.H. (2009). Microglial physiology: Unique stimuli, specialized responses. Annu. Rev. Immunol..

[75] Aderem A., Underhill D.M. (1999). Mechanisms of phagocytosis in macrophages. Annu. Rev. Immunol..

[76] Sierra A., Encinas J.M., Deudero J.J., Chancey J.H., Enikolopov G., Overstreet-Wadiche L.S., Tsirka S.E., Maletic-Savatic M. (2010). Microglia shape adult hippocampal neurogenesis through apoptosis-coupled phagocytosis. Cell Stem Cell.

[77] Savill J., Dransfield I., Gregory C., Haslett C. (2002). A blast from the past: Clearance of apoptotic cells regulates immune responses. Nat. Rev. Immunol..

[78] Dzwonek J., Rylski M., Kaczmarek L. (2004). Matrix metalloproteinases and their endogenous inhibitors in neuronal physiology of the adult brain. FEBS Lett..

[79] Baranes D., Lederfein D., Huang Y.Y., Chen M., Bailey C.H., Kandel E.R. (1998). Tissue plasminogen activator contributes to the late phase of LTP and to synaptic growth in the hippocampal mossy fiber pathway. Neuron.

[80] Ji K., Miyauchi J., Tsirka S.E. (2013). Microglia: An active player in the regulation of synaptic activity. Neural Plast..

[81] Noda M., Suzumura A. (2012). Sweepers in the CNS: Microglial Migration and Phagocytosis in the Alzheimer Disease Pathogenesis. Int. J. Alzheimers Dis..

[82] Lauber K., Blumenthal S.G., Waibel M., Wesselborg S. (2004). Clearance of apoptotic cells: Getting rid of the corpses. Mol. Cell.

[83] Brown G.C., Neher J.J. (2012). Eaten alive! Cell death by primary phagocytosis: ‘phagoptosis’. Trends Biochem. Sci..

[84] Sierra A., Abiega O., Shahraz A., Neumann H. (2013). Janus-faced microglia: Beneficial and detrimental consequences of microglial phagocytosis. Front. Cell Neurosci..

[85] Wink D.A., Hines H.B., Cheng R.Y., Switzer C.H., Flores-Santana W., Vitek M.P., Ridnour L.A., Colton C.A. (2011). Nitric oxide and redox mechanisms in the immune response. J. Leukoc. Biol..

[86] Altman J. (1969). Autoradiographic and histological studies of postnatal neurogenesis. IV. Cell proliferation and migration in the anterior forebrain, with special reference to persisting neurogenesis in the olfactory bulb. J. Comp. Neurol..

[87] Eriksson P.S., Perfilieva E., Bjork-Eriksson T., Alborn A.M., Nordborg C., Peterson D.A., Gage F.H. (1998). Neurogenesis in the adult human hippocampus. Nat. Med..

[88] Cameron H.A., McKay R. (1998). Stem cells and neurogenesis in the adult brain. Curr. Opin. Neurobiol..

[89] Ma D.K., Marchetto M.C., Guo J.U., Ming G.L., Gage F.H., Song H. (2010). Epigenetic choreographers of neurogenesis in the adult mammalian brain. Nat. Neurosci..

[90] Goncalves J.T., Schafer S.T., Gage F.H. (2016). Adult Neurogenesis in the Hippocampus: From Stem Cells to Behavior. Cell.

[91] Li G., Fang L., Fernandez G., Pleasure S.J. (2013). The ventral hippocampus is the embryonic origin for adult neural stem cells in the dentate gyrus. Neuron.

[92] Yao P.J., Petralia R.S., Mattson M.P. (2016). Sonic Hedgehog Signaling and Hippocampal Neuroplasticity. Trends Neurosci..

[93] Richards L.J., Kilpatrick T.J., Bartlett P.F. (1992). De novo generation of neuronal cells from the adult mouse brain. Proc. Natl. Acad. Sci. USA.

[94] Watt F.M., Hogan B.L. (2000). Out of Eden: Stem cells and their niches. Science.

[95] Berg D.A., Bond A.M., Ming G.L., Song H. (2018). Radial glial cells in the adult dentate gyrus: What are they and where do they come from?. F1000Research.

[96] Niu W., Zou Y., Shen C., Zhang C.L. (2011). Activation of postnatal neural stem cells requires nuclear receptor TLX. J. Neurosci..

[97] Varela-Nallar L., Inestrosa N.C. (2013). Wnt signaling in the regulation of adult hippocampal neurogenesis. Front. Cell Neurosci..

[98] van Praag H., Schinder A.F., Christie B.R., Toni N., Palmer T.D., Gage F.H. (2002). Functional neurogenesis in the adult hippocampus. Nature.

[99] Cameron H.A., McKay R.D. (2001). Adult neurogenesis produces a large pool of new granule cells in the dentate gyrus. J. Comp. Neurol..

[100] Winner B., Cooper-Kuhn C.M., Aigner R., Winkler J., Kuhn H.G. (2002). Long-term survival and cell death of newly generated neurons in the adult rat olfactory bulb. Eur. J. Neurosci..

[101] Bergmann O., Liebl J., Bernard S., Alkass K., Yeung M.S., Steier P., Kutschera W., Johnson L., Landen M., Druid H. (2012). The age of olfactory bulb neurons in humans. Neuron.

[102] Spalding K.L., Bergmann O., Alkass K., Bernard S., Salehpour M., Huttner H.B., Bostrom E., Westerlund I., Vial C., Buchholz B.A. (2013). Dynamics of hippocampal neurogenesis in adult humans. Cell.

[103] Ninkovic J., Mori T., Gotz M. (2007). Distinct modes of neuron addition in adult mouse neurogenesis. J. Neurosci..

[104] Imayoshi I., Sakamoto M., Ohtsuka T., Takao K., Miyakawa T., Yamaguchi M., Mori K., Ikeda T., Itohara S., Kageyama R. (2008). Roles of continuous neurogenesis in the structural and functional integrity of the adult forebrain. Nat. Neurosci..

[105] Kornack D.R., Rakic P. (2001). The generation, migration, and differentiation of olfactory neurons in the adult primate brain. Proc. Natl. Acad. Sci. USA.

[106] Parras C.M., Galli R., Britz O., Soares S., Galichet C., Battiste J., Johnson J.E., Nakafuku M., Vescovi A., Guillemot F. (2004). Mash1 specifies neurons and oligodendrocytes in the postnatal brain. EMBO J..

[107] Paez-Gonzalez P., Asrican B., Rodriguez E., Kuo C.T. (2014). Identification of distinct ChAT(+) neurons and activity-dependent control of postnatal SVZ neurogenesis. Nat. Neurosci..

[108] Mak G.K., Enwere E.K., Gregg C., Pakarainen T., Poutanen M., Huhtaniemi I., Weiss S. (2007). Male pheromone-stimulated neurogenesis in the adult female brain: Possible role in mating behavior. Nat. Neurosci..

[109] Mak G.K., Weiss S. (2010). Paternal recognition of adult offspring mediated by newly generated CNS neurons. Nat. Neurosci..

[110] Zhao M., Momma S., Delfani K., Carlen M., Cassidy R.M., Johansson C.B., Brismar H., Shupliakov O., Frisen J., Janson A.M. (2003). Evidence for neurogenesis in the adult mammalian substantia nigra. Proc. Natl. Acad. Sci. USA.

[111] Hitoshi S., Alexson T., Tropepe V., Donoviel D., Elia A.J., Nye J.S., Conlon R.A., Mak T.W., Bernstein A., van der Kooy D. (2002). Notch pathway molecules are essential for the maintenance, but not the generation, of mammalian neural stem cells. Genes Dev..

[112] Breunig J.J., Silbereis J., Vaccarino F.M., Sestan N., Rakic P. (2007). Notch regulates cell fate and dendrite morphology of newborn neurons in the postnatal dentate gyrus. Proc. Natl. Acad. Sci. USA.

[113] Ehm O., Goritz C., Covic M., Schaffner I., Schwarz T.J., Karaca E., Kempkes B., Kremmer E., Pfrieger F.W., Espinosa L. (2010). RBPJkappa-dependent signaling is essential for long-term maintenance of neural stem cells in the adult hippocampus. J. Neurosci..

[114] Imayoshi I., Sakamoto M., Yamaguchi M., Mori K., Kageyama R. (2010). Essential roles of Notch signaling in maintenance of neural stem cells in developing and adult brains. J. Neurosci..

[115] Lai K., Kaspar B.K., Gage F.H., Schaffer D.V. (2003). Sonic hedgehog regulates adult neural progenitor proliferation in vitro and in vivo. Nat. Neurosci..

[116] Antonelli F., Casciati A., Pazzaglia S. (2019). Sonic hedgehog signaling controls dentate gyrus patterning and adult neurogenesis in the hippocampus. Neural Regen. Res..

[117] Bond A.M., Peng C.Y., Meyers E.A., McGuire T., Ewaleifoh O., Kessler J.A. (2014). BMP signaling regulates the tempo of adult hippocampal progenitor maturation at multiple stages of the lineage. Stem Cells.

[118] Lim D.A., Tramontin A.D., Trevejo J.M., Herrera D.G., Garcia-Verdugo J.M., Alvarez-Buylla A. (2000). Noggin antagonizes BMP signaling to create a niche for adult neurogenesis. Neuron.

[119] Habas R., Dawid I.B. (2005). Dishevelled and Wnt signaling: Is the nucleus the final frontier?. J. Biol..

[120] Hermann D.M., ElAli A. (2012). The abluminal endothelial membrane in neurovascular remodeling in health and disease. Sci. Signal..

[121] Ferkey D.M., Kimelman D. (2000). GSK-3: New thoughts on an old enzyme. Dev. Biol..

[122] He P., Shen Y. (2009). Interruption of beta-catenin signaling reduces neurogenesis in Alzheimer’s disease. J. Neurosci..

[123] Kohn A.D., Moon R.T. (2005). Wnt and calcium signaling: Beta-catenin-independent pathways. Cell Calcium.

[124] Lie D.C., Colamarino S.A., Song H.J., Desire L., Mira H., Consiglio A., Lein E.S., Jessberger S., Lansford H., Dearie A.R. (2005). Wnt signalling regulates adult hippocampal neurogenesis. Nature.

[125] Kuwabara T., Hsieh J., Muotri A., Yeo G., Warashina M., Lie D.C., Moore L., Nakashima K., Asashima M., Gage F.H. (2009). Wnt-mediated activation of NeuroD1 and retro-elements during adult neurogenesis. Nat. Neurosci..

[126] Gao Z., Ure K., Ables J.L., Lagace D.C., Nave K.A., Goebbels S., Eisch A.J., Hsieh J. (2009). Neurod1 is essential for the survival and maturation of adult-born neurons. Nat. Neurosci..

[127] Kawano Y., Kypta R. (2003). Secreted antagonists of the Wnt signalling pathway. J. Cell Sci..

[128] Seib D.R., Corsini N.S., Ellwanger K., Plaas C., Mateos A., Pitzer C., Niehrs C., Celikel T., Martin-Villalba A. (2013). Loss of Dickkopf-1 restores neurogenesis in old age and counteracts cognitive decline. Cell Stem Cell.

[129] Yao B., Jin P. (2014). Unlocking epigenetic codes in neurogenesis. Genes Dev..

[130] Goto K., Numata M., Komura J.I., Ono T., Bestor T.H., Kondo H. (1994). Expression of DNA methyltransferase gene in mature and immature neurons as well as proliferating cells in mice. Differentiation.

[131] Noguchi H., Kimura A., Murao N., Namihira M., Nakashima K. (2016). Prenatal deletion of DNA methyltransferase 1 in neural stem cells impairs neurogenesis and causes anxiety-like behavior in adulthood. Neurogenesis (Austin).

[132] Fan G., Martinowich K., Chin M.H., He F., Fouse S.D., Hutnick L., Hattori D., Ge W., Shen Y., Wu H. (2005). DNA methylation controls the timing of astrogliogenesis through regulation of JAK-STAT signaling. Development.

[133] Feng J., Zhou Y., Campbell S.L., Le T., Li E., Sweatt J.D., Silva A.J., Fan G. (2010). Dnmt1 and Dnmt3a maintain DNA methylation and regulate synaptic function in adult forebrain neurons. Nat. Neurosci..

[134] Wu H., Coskun V., Tao J., Xie W., Ge W., Yoshikawa K., Li E., Zhang Y., Sun Y.E. (2010). Dnmt3a-dependent nonpromoter DNA methylation facilitates transcription of neurogenic genes. Science.

[135] Veremeyko T., Yung A.W.Y., Dukhinova M., Strekalova T., Ponomarev E.D. (2019). The Role of Neuronal Factors in the Epigenetic Reprogramming of Microglia in the Normal and Diseased Central Nervous System. Front. Cell Neurosci..

[136] Choi K.Y., Yoo M., Han J.H. (2015). Toward understanding the role of the neuron-specific BAF chromatin remodeling complex in memory formation. Exp. Mol. Med..

[137] Satoh T., Takeuchi O., Vandenbon A., Yasuda K., Tanaka Y., Kumagai Y., Miyake T., Matsushita K., Okazaki T., Saitoh T. (2010). The Jmjd3-Irf4 axis regulates M2 macrophage polarization and host responses against helminth infection. Nat. Immunol..

[138] Buttgereit A., Lelios I., Yu X., Vrohlings M., Krakoski N.R., Gautier E.L., Nishinakamura R., Becher B., Greter M. (2016). Sall1 is a transcriptional regulator defining microglia identity and function. Nat. Immunol..

[139] Veremeyko T., Yung A.W.Y., Anthony D.C., Strekalova T., Ponomarev E.D. (2018). Early Growth Response Gene-2 Is Essential for M1 and M2 Macrophage Activation and Plasticity by Modulation of the Transcription Factor CEBPbeta. Front. Immunol..

[140] Harrison S.J., Nishinakamura R., Jones K.R., Monaghan A.P. (2012). Sall1 regulates cortical neurogenesis and laminar fate specification in mice: Implications for neural abnormalities in Townes-Brocks syndrome. Dis. Model. Mech..

[141] Colonna M., Butovsky O. (2017). Microglia Function in the Central Nervous System During Health and Neurodegeneration. Annu. Rev. Immunol..

[142] Mosher K.I., Andres R.H., Fukuhara T., Bieri G., Hasegawa-Moriyama M., He Y., Guzman R., Wyss-Coray T. (2012). Neural progenitor cells regulate microglia functions and activity. Nat. Neurosci..

[143] Walton N.M., Sutter B.M., Laywell E.D., Levkoff L.H., Kearns S.M., Marshall G.P., Scheffler B., Steindler D.A. (2006). Microglia instruct subventricular zone neurogenesis. Glia.

[144] Rodriguez-Iglesias N., Sierra A., Valero J. (2019). Rewiring of Memory Circuits: Connecting Adult Newborn Neurons With the Help of Microglia. Front. Cell Dev. Biol..

[145] Aarum J., Sandberg K., Haeberlein S.L., Persson M.A. (2003). Migration and differentiation of neural precursor cells can be directed by microglia. Proc. Natl. Acad. Sci. USA.

[146] Takahashi K., Kakuda Y., Munemoto S., Yamazaki H., Nozaki I., Yamada M. (2015). Differentiation of Donor-Derived Cells Into Microglia After Umbilical Cord Blood Stem Cell Transplantation. J. Neuropathol. Exp. Neurol..

[147] Ribeiro Xavier A.L., Kress B.T., Goldman S.A., Lacerda de Menezes J.R., Nedergaard M. (2015). A Distinct Population of Microglia Supports Adult Neurogenesis in the Subventricular Zone. J. Neurosci..

[148] Kyle J., Wu M., Gourzi S., Tsirka S.E. (2019). Proliferation and Differentiation in the Adult Subventricular Zone Are Not Affected by CSF1R Inhibition. Front. Cell Neurosci..

[149] Reshef R., Kudryavitskaya E., Shani-Narkiss H., Isaacson B., Rimmerman N., Mizrahi A., Yirmiya R. (2017). The role of microglia and their CX3CR1 signaling in adult neurogenesis in the olfactory bulb. elife.

[150] Sierra A., Beccari S., Diaz-Aparicio I., Encinas J.M., Comeau S., Tremblay M.E. (2014). Surveillance, phagocytosis, and inflammation: How never-resting microglia influence adult hippocampal neurogenesis. Neural Plast..

[151] Ziv Y., Ron N., Butovsky O., Landa G., Sudai E., Greenberg N., Cohen H., Kipnis J., Schwartz M. (2006). Immune cells contribute to the maintenance of neurogenesis and spatial learning abilities in adulthood. Nat. Neurosci..

[152] Shigemoto-Mogami Y., Hoshikawa K., Goldman J.E., Sekino Y., Sato K. (2014). Microglia enhance neurogenesis and oligodendrogenesis in the early postnatal subventricular zone. J. Neurosci..

[153] Ekdahl C.T., Kokaia Z., Lindvall O. (2009). Brain inflammation and adult neurogenesis: The dual role of microglia. Neuroscience.

[154] Cacci E., Ajmone-Cat M.A., Anelli T., Biagioni S., Minghetti L. (2008). In vitro neuronal and glial differentiation from embryonic or adult neural precursor cells are differently affected by chronic or acute activation of microglia. Glia.

[155] Li L., Walker T.L., Zhang Y., Mackay E.W., Bartlett P.F. (2010). Endogenous interferon gamma directly regulates neural precursors in the non-inflammatory brain. J. Neurosci..

[156] Butovsky O., Ziv Y., Schwartz A., Landa G., Talpalar A.E., Pluchino S., Martino G., Schwartz M. (2006). Microglia activated by IL-4 or IFN-gamma differentially induce neurogenesis and oligodendrogenesis from adult stem/progenitor cells. Mol. Cell Neurosci..

[157] Diaz-Aparicio I., Paris I., Sierra-Torre V., Plaza-Zabala A., Rodriguez-Iglesias N., Marquez-Ropero M., Beccari S., Huguet P., Abiega O., Alberdi E. (2020). Microglia Actively Remodel Adult Hippocampal Neurogenesis through the Phagocytosis Secretome. J. Neurosci..

[158] Naik S., Larsen S.B., Cowley C.J., Fuchs E. (2018). Two to Tango: Dialog between Immunity and Stem Cells in Health and Disease. Cell.

[159] Mo M., Eyo U.B., Xie M., Peng J., Bosco D.B., Umpierre A.D., Zhu X., Tian D.S., Xu P., Wu L.J. (2019). Microglial P2Y12 Receptor Regulates Seizure-Induced Neurogenesis and Immature Neuronal Projections. J. Neurosci..

[160] Bachstetter A.D., Morganti J.M., Jernberg J., Schlunk A., Mitchell S.H., Brewster K.W., Hudson C.E., Cole M.J., Harrison J.K., Bickford P.C. (2011). Fractalkine and CX 3 CR1 regulate hippocampal neurogenesis in adult and aged rats. Neurobiol. Aging.

[161] Holtmaat A., Wilbrecht L., Knott G.W., Welker E., Svoboda K. (2006). Experience-dependent and cell-type-specific spine growth in the neocortex. Nature.

[162] Vukovic J., Colditz M.J., Blackmore D.G., Ruitenberg M.J., Bartlett P.F. (2012). Microglia modulate hippocampal neural precursor activity in response to exercise and aging. J. Neurosci..

[163] van Praag H., Kempermann G., Gage F.H. (1999). Running increases cell proliferation and neurogenesis in the adult mouse dentate gyrus. Nat. Neurosci..

[164] Zang J., Liu Y., Li W., Xiao D., Zhang Y., Luo Y., Liang W., Liu F., Wei W. (2017). Voluntary exercise increases adult hippocampal neurogenesis by increasing GSK-3beta activity in mice. Neuroscience.

[165] Yousef H., Morgenthaler A., Schlesinger C., Bugaj L., Conboy I.M., Schaffer D.V. (2015). Age-Associated Increase in BMP Signaling Inhibits Hippocampal Neurogenesis. Stem Cells.

[166] Meyers E.A., Gobeske K.T., Bond A.M., Jarrett J.C., Peng C.Y., Kessler J.A. (2016). Increased bone morphogenetic protein signaling contributes to age-related declines in neurogenesis and cognition. Neurobiol. Aging.

[167] Nithianantharajah J., Hannan A.J. (2006). Enriched environments, experience-dependent plasticity and disorders of the nervous system. Nat. Rev. Neurosci.

[168] Kempermann G., Brandon E.P., Gage F.H. (1998). Environmental stimulation of 129/SvJ mice causes increased cell proliferation and neurogenesis in the adult dentate gyrus. Curr. Biol..

[169] Lee T.H., Formolo D.A., Kong T., Lau S.W., Ho C.S., Leung R.Y.H., Hung F.H., Yau S.Y. (2019). Potential exerkines for physical exercise-elicited pro-cognitive effects: Insight from clinical and animal research. Int. Rev. Neurobiol..

[170] van Praag H., Shubert T., Zhao C., Gage F.H. (2005). Exercise enhances learning and hippocampal neurogenesis in aged mice. J. Neurosci..

[171] Nichol K.E., Poon W.W., Parachikova A.I., Cribbs D.H., Glabe C.G., Cotman C.W. (2008). Exercise alters the immune profile in Tg2576 Alzheimer mice toward a response coincident with improved cognitive performance and decreased amyloid. J. Neuroinflamm..

[172] Kohman R.A., DeYoung E.K., Bhattacharya T.K., Peterson L.N., Rhodes J.S. (2012). Wheel running attenuates microglia proliferation and increases expression of a proneurogenic phenotype in the hippocampus of aged mice. Brain Behav. Immun..

[173] Gray S.C., Kinghorn K.J., Woodling N.S. (2020). Shifting equilibriums in Alzheimer’s disease: The complex roles of microglia in neuroinflammation, neuronal survival and neurogenesis. Neural Regen. Res..

[174] De Lucia C., Rinchon A., Olmos-Alonso A., Riecken K., Fehse B., Boche D., Perry V.H., Gomez-Nicola D. (2016). Microglia regulate hippocampal neurogenesis during chronic neurodegeneration. Brain Behav. Immun..

[175] Battista D., Ferrari C.C., Gage F.H., Pitossi F.J. (2006). Neurogenic niche modulation by activated microglia: Transforming growth factor beta increases neurogenesis in the adult dentate gyrus. Eur. J. Neurosci..

[176] Wachs F.P., Winner B., Couillard-Despres S., Schiller T., Aigner R., Winkler J., Bogdahn U., Aigner L. (2006). Transforming growth factor-beta1 is a negative modulator of adult neurogenesis. J. Neuropathol. Exp. Neurol..

[177] Monji A., Kato T., Kanba S. (2009). Cytokines and schizophrenia: Microglia hypothesis of schizophrenia. Psychiatry Clin. Neurosci..

[178] Ekdahl C.T., Claasen J.H., Bonde S., Kokaia Z., Lindvall O. (2003). Inflammation is detrimental for neurogenesis in adult brain. Proc. Natl. Acad. Sci. USA.

[179] Monje M.L., Toda H., Palmer T.D. (2003). Inflammatory blockade restores adult hippocampal neurogenesis. Science.

[180] Tyrtyshnaia A., Manzhulo I., Kipryushina Y., Ermolenko E. (2019). Neuroinflammation and adult hippocampal neurogenesis in neuropathic pain and alkyl glycerol ethers treatment in aged mice. Int. J. Mol. Med..

[181] Seaman M.N. (2012). The retromer complex—endosomal protein recycling and beyond. J. Cell Sci..

[182] Lucin K.M., O’Brien C.E., Bieri G., Czirr E., Mosher K.I., Abbey R.J., Mastroeni D.F., Rogers J., Spencer B., Masliah E. (2013). Microglial beclin 1 regulates retromer trafficking and phagocytosis and is impaired in Alzheimer’s disease. Neuron.

[183] Appel J.R., Ye S., Tang F., Sun D., Zhang H., Mei L., Xiong W.C. (2018). Increased Microglial Activity, Impaired Adult Hippocampal Neurogenesis, and Depressive-like Behavior in Microglial VPS35-Depleted Mice. J. Neurosci..

[184] Yang Y., Zhang M., Kang X., Jiang C., Zhang H., Wang P., Li J. (2015). Thrombin-induced microglial activation impairs hippocampal neurogenesis and spatial memory ability in mice. Behav. Brain Funct..

[185] Kempermann G., Kuhn H.G., Gage F.H. (1998). Experience-induced neurogenesis in the senescent dentate gyrus. J. Neurosci..

[186] Lee S.W., Clemenson G.D., Gage F.H. (2012). New neurons in an aged brain. Behav Brain Res..

[187] Walter J., Keiner S., Witte O.W., Redecker C. (2011). Age-related effects on hippocampal precursor cell subpopulations and neurogenesis. Neurobiol. Aging.

[188] Kozareva D.A., Cryan J.F., Nolan Y.M. (2019). Born this way: Hippocampal neurogenesis across the lifespan. Aging Cell.

[189] Kempermann G., Gage F.H., Aigner L., Song H., Curtis M.A., Thuret S., Kuhn H.G., Jessberger S., Frankland P.W., Cameron H.A. (2018). Human Adult Neurogenesis: Evidence and Remaining Questions. Cell Stem Cell.

[190] Sorrells S.F., Paredes M.F., Cebrian-Silla A., Sandoval K., Qi D., Kelley K.W., James D., Mayer S., Chang J., Auguste K.I. (2018). Human hippocampal neurogenesis drops sharply in children to undetectable levels in adults. Nature.

[191] Boldrini M., Fulmore C.A., Tartt A.N., Simeon L.R., Pavlova I., Poposka V., Rosoklija G.B., Stankov A., Arango V., Dwork A.J. (2018). Human Hippocampal Neurogenesis Persists throughout Aging. Cell Stem Cell.

[192] Hefendehl J.K., Neher J.J., Suhs R.B., Kohsaka S., Skodras A., Jucker M. (2014). Homeostatic and injury-induced microglia behavior in the aging brain. Aging Cell.

[193] Sierra A., Gottfried-Blackmore A.C., McEwen B.S., Bulloch K. (2007). Microglia derived from aging mice exhibit an altered inflammatory profile. Glia.

[194] Pluvinage J.V., Haney M.S., Smith B.A.H., Sun J., Iram T., Bonanno L., Li L., Lee D.P., Morgens D.W., Yang A.C. (2019). CD22 blockade restores homeostatic microglial phagocytosis in ageing brains. Nature.

[195] Safaiyan S., Kannaiyan N., Snaidero N., Brioschi S., Biber K., Yona S., Edinger A.L., Jung S., Rossner M.J., Simons M. (2016). Age-related myelin degradation burdens the clearance function of microglia during aging. Nat. Neurosci..

[196] Song G.J., Suk K. (2017). Pharmacological Modulation of Functional Phenotypes of Microglia in Neurodegenerative Diseases. Front. Aging Neurosci..

[197] Norden D.M., Godbout J.P. (2013). Review: Microglia of the aged brain: Primed to be activated and resistant to regulation. Neuropathol. Appl. Neurobiol..

[198] Dilger R.N., Johnson R.W. (2008). Aging, microglial cell priming, and the discordant central inflammatory response to signals from the peripheral immune system. J. Leukoc. Biol..

[199] Biscaro B., Lindvall O., Tesco G., Ekdahl C.T., Nitsch R.M. (2012). Inhibition of microglial activation protects hippocampal neurogenesis and improves cognitive deficits in a transgenic mouse model for Alzheimer’s disease. Neurodegener. Dis..

[200] Rogers J.T., Morganti J.M., Bachstetter A.D., Hudson C.E., Peters M.M., Grimmig B.A., Weeber E.J., Bickford P.C., Gemma C. (2011). CX3CR1 deficiency leads to impairment of hippocampal cognitive function and synaptic plasticity. J. Neurosci..

[201] Gemma C., Bachstetter A.D., Bickford P.C. (2010). Neuron-Microglia Dialogue and Hippocampal Neurogenesis in the Aged Brain. Aging Dis..

[202] Lee S., Varvel N.H., Konerth M.E., Xu G., Cardona A.E., Ransohoff R.M., Lamb B.T. (2010). CX3CR1 deficiency alters microglial activation and reduces beta-amyloid deposition in two Alzheimer’s disease mouse models. Am. J. Pathol..

[203] Liu Z., Condello C., Schain A., Harb R., Grutzendler J. (2010). CX3CR1 in microglia regulates brain amyloid deposition through selective protofibrillar amyloid-beta phagocytosis. J. Neurosci..

[204] Hickman S.E., Allison E.K., Coleman U., Kingery-Gallagher N.D., El Khoury J. (2019). Heterozygous CX3CR1 Deficiency in Microglia Restores Neuronal beta-Amyloid Clearance Pathways and Slows Progression of Alzheimer’s Like-Disease in PS1-APP Mice. Front. Immunol..

[205] Selkoe D.J. (2011). Alzheimer’s disease. Cold Spring Harb Perspect Biol..

[206] Jack C.R., Knopman D.S., Jagust W.J., Petersen R.C., Weiner M.W., Aisen P.S., Shaw L.M., Vemuri P., Wiste H.J., Weigand S.D. (2013). Tracking pathophysiological processes in Alzheimer’s disease: An updated hypothetical model of dynamic biomarkers. Lancet Neurol..

[207] Haass C., Selkoe D.J. (2007). Soluble protein oligomers in neurodegeneration: Lessons from the Alzheimer’s amyloid beta-peptide. Nat. Rev. Mol. Cell Biol..

[208] Scopa C., Marrocco F., Latina V., Ruggeri F., Corvaglia V., La Regina F., Ammassari-Teule M., Middei S., Amadoro G., Meli G. (2020). Correction to: Impaired adult neurogenesis is an early event in Alzheimer’s disease neurodegeneration, mediated by intracellular Abeta oligomers. Cell Death Differ..

[209] Sierra A., Encinas J.M., Maletic-Savatic M. (2011). Adult human neurogenesis: From microscopy to magnetic resonance imaging. Front. Neurosci..

[210] Moreno-Jimenez E.P., Flor-Garcia M., Terreros-Roncal J., Rabano A., Cafini F., Pallas-Bazarra N., Avila J., Llorens-Martin M. (2019). Adult hippocampal neurogenesis is abundant in neurologically healthy subjects and drops sharply in patients with Alzheimer’s disease. Nat. Med..

[211] Tobin M.K., Musaraca K., Disouky A., Shetti A., Bheri A., Honer W.G., Kim N., Dawe R.J., Bennett D.A., Arfanakis K. (2019). Human Hippocampal Neurogenesis Persists in Aged Adults and Alzheimer’s Disease Patients. Cell Stem Cell.

[212] Choi S.H., Veeraraghavalu K., Lazarov O., Marler S., Ransohoff R.M., Ramirez J.M., Sisodia S.S. (2008). Non-cell-autonomous effects of presenilin 1 variants on enrichment-mediated hippocampal progenitor cell proliferation and differentiation. Neuron.

[213] Chevallier N.L., Soriano S., Kang D.E., Masliah E., Hu G., Koo E.H. (2005). Perturbed neurogenesis in the adult hippocampus associated with presenilin-1 A246E mutation. Am. J. Pathol..

[214] Haughey N.J., Nath A., Chan S.L., Borchard A.C., Rao M.S., Mattson M.P. (2002). Disruption of neurogenesis by amyloid beta-peptide, and perturbed neural progenitor cell homeostasis, in models of Alzheimer’s disease. J. Neurochem..

[215] Jin K., Galvan V., Xie L., Mao X.O., Gorostiza O.F., Bredesen D.E., Greenberg D.A. (2004). Enhanced neurogenesis in Alzheimer’s disease transgenic (PDGF-APPSw,Ind) mice. Proc. Natl. Acad. Sci. USA.

[216] Jin K., Peel A.L., Mao X.O., Xie L., Cottrell B.A., Henshall D.C., Greenberg D.A. (2004). Increased hippocampal neurogenesis in Alzheimer’s disease. Proc. Natl. Acad. Sci. USA.

[217] Donovan M.H., Yazdani U., Norris R.D., Games D., German D.C., Eisch A.J. (2006). Decreased adult hippocampal neurogenesis in the PDAPP mouse model of Alzheimer’s disease. J. Comp. Neurol..

[218] Sotthibundhu A., Li Q.X., Thangnipon W., Coulson E.J. (2009). Abeta(1-42) stimulates adult SVZ neurogenesis through the p75 neurotrophin receptor. Neurobiol. Aging.

[219] Rodriguez J.J., Jones V.C., Tabuchi M., Allan S.M., Knight E.M., LaFerla F.M., Oddo S., Verkhratsky A. (2008). Impaired adult neurogenesis in the dentate gyrus of a triple transgenic mouse model of Alzheimer’s disease. PLoS ONE.

[220] Hamilton L.K., Aumont A., Julien C., Vadnais A., Calon F., Fernandes K.J. (2010). Widespread deficits in adult neurogenesis precede plaque and tangle formation in the 3xTg mouse model of Alzheimer’s disease. Eur. J. Neurosci..

[221] Myhre C.L., Thygesen C., Villadsen B., Vollerup J., Ilkjaer L., Krohn K.T., Grebing M., Zhao S., Khan A.M., Dissing-Olesen L. (2019). Microglia Express Insulin-Like Growth Factor-1 in the Hippocampus of Aged APPswe/PS1DeltaE9 Transgenic Mice. Front. Cell Neurosci..

[222] Tesseur I., Zou K., Esposito L., Bard F., Berber E., Can J.V., Lin A.H., Crews L., Tremblay P., Mathews P. (2006). Deficiency in neuronal TGF-beta signaling promotes neurodegeneration and Alzheimer’s pathology. J. Clin. Investig..

[223] Lee M.S., Tsai L.H. (2003). Cdk5: One of the links between senile plaques and neurofibrillary tangles?. J. Alzheimers Dis..

[224] Demars M., Hu Y.S., Gadadhar A., Lazarov O. (2010). Impaired neurogenesis is an early event in the etiology of familial Alzheimer’s disease in transgenic mice. J. Neurosci. Res..

[225] Sanchez-Mejias E., Navarro V., Jimenez S., Sanchez-Mico M., Sanchez-Varo R., Nunez-Diaz C., Trujillo-Estrada L., Davila J.C., Vizuete M., Gutierrez A. (2016). Soluble phospho-tau from Alzheimer’s disease hippocampus drives microglial degeneration. Acta Neuropathol..

[226] Vogels T., Murgoci A.N., Hromadka T. (2019). Intersection of pathological tau and microglia at the synapse. Acta Neuropathol. Commun..

[227] Bellucci A., Westwood A.J., Ingram E., Casamenti F., Goedert M., Spillantini M.G. (2004). Induction of inflammatory mediators and microglial activation in mice transgenic for mutant human P301S tau protein. Am. J. Pathol..

[228] Laurent C., Dorothee G., Hunot S., Martin E., Monnet Y., Duchamp M., Dong Y., Legeron F.P., Leboucher A., Burnouf S. (2017). Hippocampal T cell infiltration promotes neuroinflammation and cognitive decline in a mouse model of tauopathy. Brain.

[229] Yoshiyama Y., Higuchi M., Zhang B., Huang S.M., Iwata N., Saido T.C., Maeda J., Suhara T., Trojanowski J.Q., Lee V.M. (2007). Synapse loss and microglial activation precede tangles in a P301S tauopathy mouse model. Neuron.

[230] Maphis N., Xu G., Kokiko-Cochran O.N., Jiang S., Cardona A., Ransohoff R.M., Lamb B.T., Bhaskar K. (2015). Reactive microglia drive tau pathology and contribute to the spreading of pathological tau in the brain. Brain.

[231] Winner B., Lie D.C., Rockenstein E., Aigner R., Aigner L., Masliah E., Kuhn H.G., Winkler J. (2004). Human wild-type alpha-synuclein impairs neurogenesis. J. Neuropathol. Exp. Neurol..

[232] Hoglinger G.U., Rizk P., Muriel M.P., Duyckaerts C., Oertel W.H., Caille I., Hirsch E.C. (2004). Dopamine depletion impairs precursor cell proliferation in Parkinson disease. Nat. Neurosci..

[233] Winner B., Regensburger M., Schreglmann S., Boyer L., Prots I., Rockenstein E., Mante M., Zhao C., Winkler J., Masliah E. (2012). Role of alpha-synuclein in adult neurogenesis and neuronal maturation in the dentate gyrus. J. Neurosci..

[234] O’Keeffe G.C., Tyers P., Aarsland D., Dalley J.W., Barker R.A., Caldwell M.A. (2009). Dopamine-induced proliferation of adult neural precursor cells in the mammalian subventricular zone is mediated through EGF. Proc. Natl. Acad. Sci. USA.

[235] Park J.H., Enikolopov G. (2010). Transient elevation of adult hippocampal neurogenesis after dopamine depletion. Exp. Neurol..

[236] Ermine C.M., Wright J.L., Frausin S., Kauhausen J.A., Parish C.L., Stanic D., Thompson L.H. (2018). Modelling the dopamine and noradrenergic cell loss that occurs in Parkinson’s disease and the impact on hippocampal neurogenesis. Hippocampus.

[237] L’Episcopo F., Tirolo C., Testa N., Caniglia S., Morale M.C., Deleidi M., Serapide M.F., Pluchino S., Marchetti B. (2012). Plasticity of subventricular zone neuroprogenitors in MPTP (1-methyl-4-phenyl-1,2,3,6-tetrahydropyridine) mouse model of Parkinson’s disease involves cross talk between inflammatory and Wnt/beta-catenin signaling pathways: Functional consequences for neuroprotection and repair. J. Neurosci..

[238] Schapira A.H., Olanow C.W., Greenamyre J.T., Bezard E. (2014). Slowing of neurodegeneration in Parkinson’s disease and Huntington’s disease: Future therapeutic perspectives. Lancet.

[239] O’Keeffe G.C., Barker R.A., Caldwell M.A. (2009). Dopaminergic modulation of neurogenesis in the subventricular zone of the adult brain. Cell Cycle.

[240] Nuber S., Petrasch-Parwez E., Winner B., Winkler J., von Horsten S., Schmidt T., Boy J., Kuhn M., Nguyen H.P., Teismann P. (2008). Neurodegeneration and motor dysfunction in a conditional model of Parkinson’s disease. J. Neurosci..

[241] Kohl Z., Winner B., Ubhi K., Rockenstein E., Mante M., Munch M., Barlow C., Carter T., Masliah E., Winkler J. (2012). Fluoxetine rescues impaired hippocampal neurogenesis in a transgenic A53T synuclein mouse model. Eur. J. Neurosci..

[242] Crews L., Mizuno H., Desplats P., Rockenstein E., Adame A., Patrick C., Winner B., Winkler J., Masliah E. (2008). Alpha-synuclein alters Notch-1 expression and neurogenesis in mouse embryonic stem cells and in the hippocampus of transgenic mice. J. Neurosci..

[243] Winner B., Winkler J. (2015). Adult neurogenesis in neurodegenerative diseases. Cold Spring Harb. Perspect. Biol..

[244] Baker S.A., Baker K.A., Hagg T. (2004). Dopaminergic nigrostriatal projections regulate neural precursor proliferation in the adult mouse subventricular zone. Eur. J. Neurosci..

[245] Winner B., Geyer M., Couillard-Despres S., Aigner R., Bogdahn U., Aigner L., Kuhn G., Winkler J. (2006). Striatal deafferentation increases dopaminergic neurogenesis in the adult olfactory bulb. Exp. Neurol..

[246] Doorn K.J., Goudriaan A., Blits-Huizinga C., Bol J.G., Rozemuller A.J., Hoogland P.V., Lucassen P.J., Drukarch B., van de Berg W.D., van Dam A.M. (2014). Increased amoeboid microglial density in the olfactory bulb of Parkinson’s and Alzheimer’s patients. Brain Pathol..

[247] Vroon A., Drukarch B., Bol J.G., Cras P., Breve J.J., Allan S.M., Relton J.K., Hoogland P.V., Van Dam A.M. (2007). Neuroinflammation in Parkinson’s patients and MPTP-treated mice is not restricted to the nigrostriatal system: Microgliosis and differential expression of interleukin-1 receptors in the olfactory bulb. Exp. Gerontol..

[248] Yang P., Arnold S.A., Habas A., Hetman M., Hagg T. (2008). Ciliary neurotrophic factor mediates dopamine D2 receptor-induced CNS neurogenesis in adult mice. J. Neurosci..

[249] Baek J.Y., Jeong J.Y., Kim K.I., Won S.Y., Chung Y.C., Nam J.H., Cho E.J., Ahn T.B., Bok E., Shin W.H. (2018). Inhibition of Microglia-Derived Oxidative Stress by Ciliary Neurotrophic Factor Protects Dopamine Neurons In Vivo from MPP(+) Neurotoxicity. Int. J. Mol. Sci..

[250] Glass C.K., Saijo K., Winner B., Marchetto M.C., Gage F.H. (2010). Mechanisms underlying inflammation in neurodegeneration. Cell.

[251] Marchetti B., Tirolo C., L’Episcopo F., Caniglia S., Testa N., Smith J.A., Pluchino S., Serapide M.F. (2020). Parkinson’s disease, aging and adult neurogenesis: Wnt/beta-catenin signalling as the key to unlock the mystery of endogenous brain repair. Aging Cell.

[252] L’Episcopo F., Tirolo C., Testa N., Caniglia S., Morale M.C., Impagnatiello F., Pluchino S., Marchetti B. (2013). Aging-induced Nrf2-ARE pathway disruption in the subventricular zone drives neurogenic impairment in parkinsonian mice via PI3K-Wnt/beta-catenin dysregulation. J. Neurosci..

[253] Mishra A., Singh S., Tiwari V., Chaturvedi S., Wahajuddin M., Shukla S. (2019). Dopamine receptor activation mitigates mitochondrial dysfunction and oxidative stress to enhance dopaminergic neurogenesis in 6-OHDA lesioned rats: A role of Wnt signalling. Neurochem. Int..

[254] Kuhn H.G. (2015). Control of Cell Survival in Adult Mammalian Neurogenesis. Cold Spring Harb. Perspect. Biol..

[255] Sung P.S., Lin P.Y., Liu C.H., Su H.C., Tsai K.J. (2020). Neuroinflammation and Neurogenesis in Alzheimer’s Disease and Potential Therapeutic Approaches. Int. J. Mol. Sci..

